# Cholangiocyte-Derived Exosomal lncRNA H19 Promotes Macrophage Activation and Hepatic Inflammation under Cholestatic Conditions

**DOI:** 10.3390/cells9010190

**Published:** 2020-01-11

**Authors:** Xiaojiaoyang Li, Runping Liu, Yanyan Wang, Weiwei Zhu, Derrick Zhao, Xuan Wang, Hang Yang, Emily C. Gurley, Weidong Chen, Phillip B. Hylemon, Huiping Zhou

**Affiliations:** 1 Department of Microbiology and Immunology and McGuire Veterans Affairs Medical Center, Virginia Commonwealth University, Richmond, VA 23298, USA; Xiaojiaoyang.Li@vcuhealth.org (X.L.); lrpscreen@live.com (R.L.); aucmwyy@hotmail.com (Y.W.); zww940716@hotmail.com (W.Z.); Derrick.Zhao@vcuhealth.org (D.Z.); xuan.wang@vcuhealth.org (X.W.); andyyang529@outlook.com (H.Y.); Emily.Gurley@vcuhealth.org (E.C.G.); Phillip.Hylemon@vcuhealth.org (P.B.H.); 2 Division of Gastroenterology, Hepatology and Nutrition and McGuire Veterans Affairs Medical Center, Virginia Commonwealth University, Richmond, VA 23298, USA; 3 School of Pharmaceutical Science, Anhui University of Chinese Medicine, Hefei 230031, China; anzhongdong@126.com

**Keywords:** long non-coding RNA H19, exosomes, macrophages, Kupffer cells, CCL-2

## Abstract

Activation of hepatic macrophages represents the critical driving force to promote cholestatic liver injury. Exosomes, as important small extracellular vesicles released by almost all types of cells, contribute to intercellular communication. We previously reported that cholangiocyte-derived exosomal long noncoding RNA (lncRNA) H19 plays a vital role in disrupting bile acid homeostasis in hepatocytes and promoting the activation of hepatic stellate cells (HSCs). Exosomal H19 derived from cholangiocytes was rapidly taken up by Kupffer cells. However, the mechanistic links between exosomal lncRNA H19 and macrophage-driven inflammation in cholestasis remain unclear. Here, we reported that the hepatic H19 level was closely correlated with macrophage activation and hepatic fibrosis in both Mdr2^-/-^ and bile duct ligation (BDL) cholestatic mouse models, as well as in human primary sclerosing cholangitis (PSC) and primary biliary cholangitis (PBC) patients. Exosomal H19 significantly induced the expression and secretion of chemokine (C–C motif) ligand 2 (CCL-2) and interleukin 6 (IL-6) in Kupffer cells. H19-enriched exosomes enhanced the activation M1 polarization of Kupffer cells and promoted the recruitment and differentiation of bone marrow-derived macrophages, which were inhibited by a CCL-2 pharmacological inhibitor. In conclusion, Cholangiocyte-derived exosomal H19 played a critical role in macrophage activation, differentiation, and chemotaxis through CCL-2/CCR-2 signaling pathways, which represent a therapeutic target for cholestatic liver diseases.

## 1. Introduction

Cholestatic liver diseases, including primary biliary cholangitis (PBC) and primary sclerosing cholangitis (PSC), are critical clinical problems worldwide [1,2,3]. Due to the limited understanding of their pathogenesis, the late diagnosis, and the lack of effective therapeutic options, liver transplantation remains the only solution for end-stage patients [4]. Cholangiocytes are the major targets in cholangiopathies [5]. We previously reported that taurocholic acid (TCA) stimulated cholangiocyte proliferation and enhanced the activation of inflammatory responses via the sphingosine 1-phosphate receptor 2 (S1PR2) signaling pathway [5,6]. Long non-coding RNA (lncRNA) H19, an imprinted and maternally expressed transcript [7], was predominantly expressed in cholangiocytes in the liver and was upregulated under cholestatic conditions. Our recent studies showed that aberrant expression of lncRNA H19 not only contributed to the bile duct ligation (BDL) and carbon tetrachloride (CCl_4_) and multidrug resistance 2 (Mdr2) deficiency-induced fibrotic liver injury but also was associated with disease progression in human PSC, PBC, and biliary atresia patients [8,9,10].

Increasing evidence demonstrated that exosomes, the small extracellular vesicles (EVs) released by almost all types of cells, including hepatocytes and cholangiocytes in the livers, play a critical role in cell-to-cell communication under normal and pathological conditions [11,12]. Exosome-mediated transfer of mRNA, miRNA, lncRNAs, proteins, and lipids is implicated in various liver diseases [13]. Most recently, we found that lncRNA H19 was an important cargo of cholangiocyte-derived exosomes in response to cholestatic injury. Cholangiocyte-derived exosomal H19 not only disrupted bile acid homeostasis by suppressing small heterodimeric partner (SHP) expression in hepatocytes [14] but also promoted the activation and proliferation of hepatic stellate cells (HSCs) [9], which resulted in the cholestatic liver injury in BDL and Mdr2^-/-^ mice. However, the role of H19 in macrophage activation under cholestatic conditions remains to be fully characterized.

Macrophages, including resident tissue macrophages and monocyte-derived recruited cells, differentiate into either classically activated M1 or alternatively activated M2 phenotypes [15,16]. Chemokine (C-C motif) ligand 2 (CCL-2) is a natural ligand for a G-protein-coupled receptor, C-C chemokine receptor type 2 (CCR-2). CCL-2 is primarily expressed in myeloid cells, including monocytes, macrophages, and dendritic cells, and CCR-2 is more broadly expressed in macrophages, lymphocytes, endothelial cells, epithelial cells, fibroblasts, and mesenchymal stem cells [17]. In the liver, in response to accumulated bile acids, resident macrophages rapidly release cytokines/chemokines, including CCL-2. Increased secretion of CCL-2 further recruits the CCR-2 expressing cells, such as pro-inflammatory monocytes, to the injured site [18,19]. Previous studies demonstrated that the inhibition of either CCL-2 or CCR-2 decreased M1 macrophage-derived cytokines and restrained the recruitment and chemotaxis of circulating monocytes [20,21]. The cholestatic liver injury was largely alleviated in CCL-2^-/-^ or CCR-2^-/-^ mice [22,23,24]. However, whether the activation of CCL-2/CCR-2 is linked to aberrant H19 expression in cholestatic injury has not been elucidated and was the focus of the current study.

Here, we demonstrated that cholangiocyte-derived H19-carrying exosomes increased CCL-2 expression in primary Kupffer cells. Exosomal H19 not only induced macrophage activation but also promoted monocyte differentiation and migration through CCL-2/CCR-2 signaling pathways. These results indicated that cholangiocyte-secreted H19-exosomes played a critical role in promoting macrophage activation and hepatic inflammation in cholestatic liver diseases.

## 2. Methods

### 2.1. Materials

The functional grade antibody against purified CCL-2 (Carlsbad, CA, USA; Cat# 16-7096-85) and purified hamster IgG control (Carlsbad, CA, USA; Cat#16-4888-85) for CCL-2 were purchased from Invitrogen (Carlsbad, CA, USA). All chemicals were purchased from Sigma-Aldrich (St. Louis, MO, USA). All cell culture medium and supplemental components were obtained from Gibco (Waltham, MA, USA). The Precision Plus Protein Kaleidoscope Standards and iQTM SYBR Green Supermix were purchased from Bio-Rad (Hercules, CA, USA). Detailed information for antibodies used in western blot analysis is provided in Supplementary Table S1.

### 2.2. Animal Studies

C57BL/6J wild type (WT) mice were purchased from Jackson Laboratories (Bar Harbor, ME, USA). Mdr2^-/-^ mice with C57BL/6J background were a gift from Dr. Daniel Goldenberg at the Department of Pathology, Hadassah-Hebrew University Medical Center, Jerusalem, Israel. Maternal H19^ΔExon1/+^ (H19KO) mice with C57BL/6J background were generated by Dr. Karl Pfeifer at NIH and provided by Dr. Jian-Ying Wang at the University of Maryland (Baltimore, MD, USA). Mdr2^-/-^H19^maternalΔExon1/+^ (DKO) mice were generated, as described previously [9]. WT, H19KO, Mdr2^-/-^ mice, and DKO mice (both male and female) at 100-day old were used. For the bile duct ligation (BDL) mouse model, WT and H19KO mice (both male and female) at 12 weeks old were subjected to 2 weeks BDL or sham operation. All mice were housed under 12 h/12 h light and dark cycle with free access to water and standard chow ad libitum. All animal experiments were performed following institutional guidelines for ethical animal studies and approved by the VCU institutional animal care and use committee (Approved protocol number: AD1001773).

### 2.3. Human Liver Samples

Frozen human liver tissues from healthy controls, PSC, and PBC patients were obtained through the liver tissue cell distribution system (Minneapolis, MN, USA), which is funded by the National Institutes of Health (contract no. HSN276201200017C).

### 2.4. Isolation and Culture of Primary Kupffer Cells and Bone Marrow-Derived Macrophages (BMDMs)

Primary mouse Kupffer cells from WT and H19KO mice were isolated using a two-step collagenase digestion method and cultured, as previously described [10]. Kupffer cells were washed with cold PBS to remove dead cells. The medium was changed every 3 days after plating. For primary BMDM isolation, mouse femurs were flushed with Dulbecco’s modified eagle medium (DMEM) with penicillin G (100 U/mL) and streptomycin (100 μg/mL) two times. Bone marrow cells were then seeded and cultured in DMEM containing 10% fetal bovine serum (FBS), penicillin G (100 U/mL), streptomycin (100 μg/mL), and 10% L-cell medium for 7 days. Adherent cells were washed and cultured with macrophage medium supplemented with 10 ng/mL granulocyte-macrophage colony-stimulating factor (GM-CSF) (differentiation medium) to allow differentiation for another 7 days.

### 2.5. Cell Culture

Mouse large cholangiocytes (MLE) were originally obtained from Dr. Gianfranco Alpini (Indiana University School of Medicine, Indianapolis, IN, USA) and were cultured with minimum essential medium (MEM) containing 10% FBS, penicillin G (100 U/mL), and streptomycin (100 μg/mL), as described previously [10]. All cells were cultured at 37 °C with 5% CO_2_ in a humidified cell culture incubator.

### 2.6. Cell Culture and Treatment

For the study of lipopolysaccharide (LPS)-induced macrophage activation, Kupffer cells were cultured and treated with control exosomes, H19-overexpression exosomes with or without LPS (10 ng/mL) for 24 h. For the study of BMDM polarization, BMDMs were cultured and differentiated for 14 days followed by treatment with M1 stimulators (LPS, 10 ng/mL and IFN-γ, 100 ng/mL) or M2 stimulators (IL-4, 20 ng/mL and IL-13, 20 ng/mL) for another 24 h. For the study of BMDM differentiation, primary BMDMs were isolated and cultured for 7 days. WT and H19KO BMDMs were cultured using fresh differentiation medium with control or H19-overexpression exosomes. The culture medium was collected on day 9 and 12. On day 14, BMDMs were treated with control or H19-overexpression exosomes and M1 stimulators for 24 h.

### 2.7. Macrophage Migration Assay

Mouse primary Kupffer cells were isolated and plated (5 × 10^4^ per well) in 24-well plates (transwell bottom chambers). BMDMs (7 × 10^5^ per well) were isolated and cultured in 6-well plates. After 5 days, BMDMs were collected and seeded in the transwell top chambers with a density of 1.2 × 10^5^ cells/well (Corning Life Science, Armonk, NY, USA). Once the BMDMs attached, top chambers were transferred into 12-well plates containing cultured primary Kupffer cells. Kupffer cells were treated with H19-carrying exosomes for 24 h with or without pretreatment of CCL-2 inhibitor Bindarit (300 μM) or functional grade purified CCL-2 antibody (20 μg/mL) or control IgG for 2 h. After incubation, a crystal violet solution was used to fix and stain migrated cells for 30 min. Non-migrating cells on the top of the transwell chamber were removed, and images were captured using Olympus microscope model DP12 (Tokyo, Japan).

### 2.8. Isolation of H19 Enriched Exosomes

MLE cells were plated on 150 mm plates and transfected with 10 μg H19 overexpression plasmid (a gift from Dr. Jianying Wang, University of Maryland School of Medicine) or control plasmid (CP) with polyJet (SignaGen Laboratories, Rockville, MD, USA) for 48 h. After treatment, exosomes from cholangiocyte cell culture medium were isolated and purified using ultracentrifugation, as described previously [14]. Pellets of exosomes were resuspended in 50 μL sterile PBS for further studies.

### 2.9. Enzyme-Linked Immunosorbent Assay (ELISA)

Mouse Kupffer cells were treated with H19-carrying exosomes and LPS (10 ng/mL) for 24 h with or without pretreatment of Bindarit (CCL-2 inhibitor, 300 μM) for 2 h. At the end of treatment, the cell culture medium was collected and centrifuged, as described previously [25]. CCL-2, TNF-α, and interleukin 6 (IL-6) levels in the media and mouse serum were determined by ELISA using mouse MCP-1, mouse TNF-α, and mouse IL-6 ELISA Max™ Set Deluxe Kits (Biolegend, San Diego, CA, USA).

### 2.10. Flow Cytometry

After a two-step collagenase digestion, hepatic non-parenchymal cells were suspended and blocked with CD16/CD32 antibody for 20 min, followed by incubation with specific fluorescence antibodies (F4/80, CD11b, and CCR-2, from Biolegend, San Diego, VA, USA) for 30 min in the dark. After washing with PBS twice, labeled single cells were stained with 100 X DAPI solution and analyzed by flow cytometry in a BD LSRFortessa-X20™ (BD Biosciences, San Jose, CA, USA) at the VCU Massey Cancer Center Flow cytometry Shared Resource (supported, in part, with funding for NIH-NCI Cancer Center Support P30 CA16059). Detailed information for antibodies used in flow cytometry analysis is provided in Supplementary Table S1.

### 2.11. Western Blot Analysis

Total cell lysate from mouse primary cells was lysed in RIPA lysis buffer (Thermo Scientific, Rockford, IL, USA). The proteins were separated by 10% SDS-PAGE electrophoresis and transferred to 0.45 µm nitrocellulose membranes (Thermo Scientific, Rockford, IL, USA). The target proteins were blotted with the indicated antibodies and detected using HRP-conjugated secondary antibodies and ECL reagents (Thermo Scientific, Rockford, IL, USA). Images were captured using the Bio-Rad Gel Doc XR+ Imaging System (Hercules, CA, USA), as described previously [26].

### 2.12. RNA Isolation and Quantitative RT-PCR

Total RNA was isolated from mouse liver tissues or cells using TRIzol Reagent (QIAGEN, Valencia, CA, USA). cDNA was reverse transcribed, and quantitative real-time RT-PCR (qPCR) was performed, as described previously [27].

### 2.13. Histopathology Staining

Mouse liver tissues were fixed with 4% formaldehyde and embedded in paraffin. Tissues were cut into 4.5-μm sections and stained with hematoxylin and eosin (H&E), as previously described [5]. Images were captured using a Zeiss Axio Scope A1 microscope with a 40× objective (Carl Zeiss, Oberkochen, Germany) at the Microscopy Shared Resource of VCU Messay Cancer Center supported partially by NIH-NCI Cancer Center Support Grant P30 CA016059.

### 2.14. Statistical Analysis

All of the experiments were repeated at least three times, and the results were expressed as the mean ± SEM (standard error of the mean). Two-tailed Student’s t-test or One-way ANOVA with Tukey’s posthoc test were performed to compare the differences between two or multiple groups using GraphPad Prism 5 software (GraphPad, San Diego, CA, USA). *p*-value ≤ 0.05 was considered statistically significant.

## 3. Results

### 3.1. Exosomal H19 from Cholangiocytes Promotes Kupffer Cell Activation

It is well established that activation of Kupffer cells and monocyte-derived macrophages result in the production of different cytokines and chemokines, which further promote the development of cholestatic liver injury [28,29]. Our recent studies showed that the aberrant expression of H19 contributed to the cholestatic liver injury in both Mdr2^-/-^ mice and BDL mice [9,10]. Emerging evidence suggests that exosomes are released from hepatocytes and nonparenchymal cells to mediate the transfer of mRNA, miRNA, lncRNA, protein, or lipids, which contribute to the progression of various liver diseases [30,31]. Most recently, we showed that the transplant of serum exosomal H19 from aged Mdr2^-/-^ mice markedly increased the levels of inflammatory cytokines and promoted cholestatic liver injury in young Mdr2^-/-^ mice [14]. In addition, H19-carrying exosomes from cholangiocytes were rapidly taken up by Kupffer cells. However, the role of cholangiocyte-derived exosomal H19 in macrophage activation and hepatic inflammation has not been studied.

**Figure 1:**
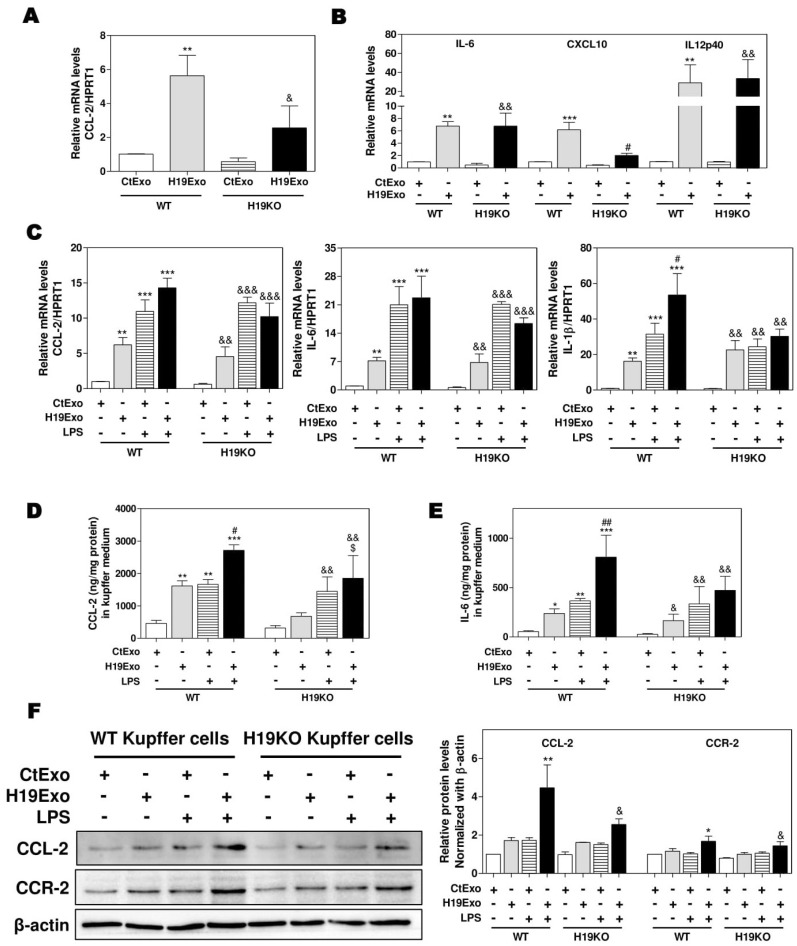
Cholangiocyte-derived exosomal H19 promotes Kupffer activation. (**A**,**B**) Mouse Kupffer cells were treated with control mouse large cholangiocytes (MLE)-derived exosomes (CtExo) and H19-overexpressing MLE-derived exosomes (H19Exo) for 24 h. (**A**,**B**) The relative mRNA levels of chemokine (C-C motif) ligand 2 (CCL-2), interleukin-6 (IL-6), chemokine (C-X-C motif) ligand 10 (CXCL10), and IL12p40 were measured by real-time RT-PCR and normalized using HPRT1 (Hypoxanthine-guanine phosphoribosyltransferase 1). (**C**–**F**) Mouse Kupffer cells were treated with CtExo, H19Exo, and lipopolysaccharide (LPS) (10 ng/mL) for 24 h. (**C**) The relative mRNA levels of CCL-2, IL-6, and IL-1β were measured by real-time RT-PCR and normalized using HPRT1. (**D**,**E**) The protein levels of CCL-2 and IL-6 in the conditioned medium of Kupffer cells were measured by ELISA assay and normalized using protein concentration. (**F**) Representative immune blot images of CCL-2 and CCR-2 are shown. Relative protein levels were normalized using β-actin. Results from at least three independent experiments are presented as Mean ± SEM. Statistical significance: * *p* < 0.05, ** *p* < 0.01, *** *p* < 0.001, compared with WT control group; ^#^ *p* < 0.05, ^##^ *p* < 0.01, compared with WT H19Exo group; ^&^ *p* < 0.05, ^&&^ *p* < 0.01, ^&&&^ *p* < 0.001, compared with H19KO control group; ^$^ *p* < 0.05, compared with H19KO H19Exo group.

To examine the effects of exosomal H19 on the activation of liver-resident macrophages, we first isolated cholangiocyte-derived exosomes from the cell culture medium of control MLE cells (CtExo) or H19 over-expressing MLE cells (H19Exo). Kupffer cells from WT and H19KO mice were treated with CtExo or H19Exo for 24 h. Interestingly, real-time PCR analysis indicated that H19Exo significantly increased the mRNA levels of CCL-2 and CCR-2, in both WT and H19KO Kupffer cells, but to a lesser extent in H19KO Kupffer cells (Figure 1A; Supplementary Figure S1). H19Exo also induced expression of M1 polarization markers, including IL-6, chemokine (C-X-C motif) ligand 10 (CXCL10), and IL12p40 (interleukin-12 subunit p40 (Figure 1B) in both WT and H19KO Kupffer cells. To further determine whether H19Exo has any effect on the bacterial endotoxin-induced inflammatory response in macrophages, WT and H19KO Kupffer cells were treated with CtExo or H19Exo without or with lipopolysaccharide (LPS). As shown in Figure 1C–E, both LPS and H19Exo increased mRNA expression levels of CCL-2, IL-6, and IL-1β in WT and H19KO Kupffer cells. LPS-induced secretion of CCL-2 and IL-6 was further enhanced by H19Exo WT Kupffer cells, but not in H19KO Kupffer cells. However, both H19Exo and LPS had similar effects on TNF-α secretion in WT and H19KO Kupffer cells (Supplementary Figure S2A). In addition, H19Exo further increased LPS-induced mRNA expression of chemokines, including CXCL10 and CCL-5, and M1 macrophage marker, IL12p40 in WT Kupffer cells, but not in H19KO Kupffer cells (Supplementary Figure S2B–D). The Western blot analysis further confirmed that H19Exo also promoted an LPS-induced increase of CCL-2 and CCR-2 protein levels in both WT and H19KO Kupffer cells, but had less impact on H19KO Kupffer cells (Figure 1F).

### 3.2. Effects of Cholangiocyte-Derived Exosomal H19 on BMDM Activation and Polarization

**Figure 2:**
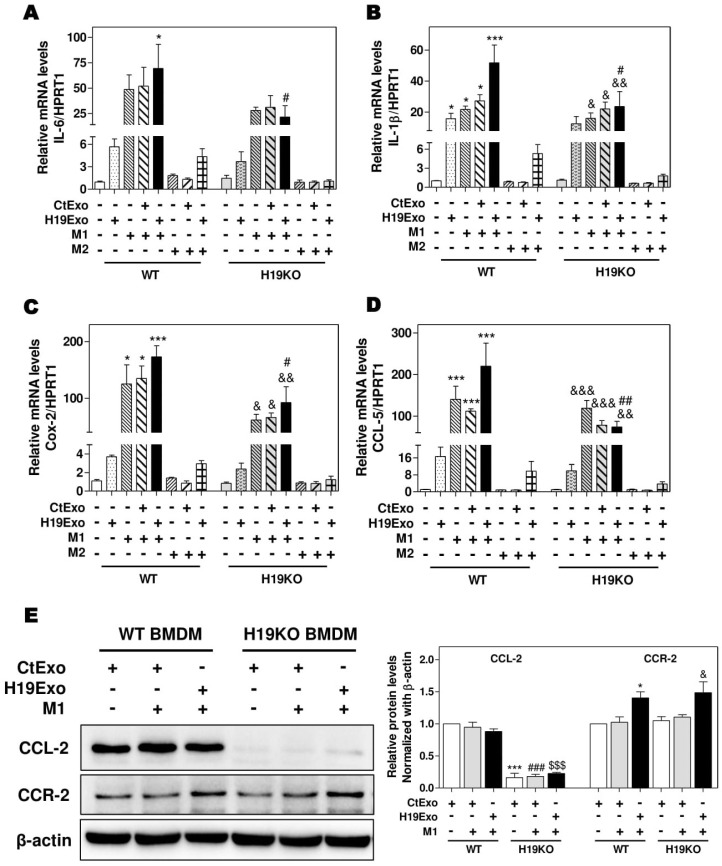
Cholangiocyte-derived exosomal H19 promotes BMDM polarization. (**A**,**B**) Mouse bone marrow-derived macrophage (BMDM) cells were treated with CtExo, H19Exo, M1 (LPS, 10 ng/mL and IFN-γ, 100 ng/mL) or M2 stimulators (IL-4, 20 ng/mL and IL-13, 20 ng/mL) for 24 h. (**A**–**D**) The relative mRNA levels of IL-6, IL-1β, Cox-2, and CCL-5 were measured by real-time RT-PCR and normalized using HPRT1. (**E**) Representative immune blot images of CCL-2 and CCR-2 are shown. Relative protein levels were normalized using β-actin. Results from at least three independent experiments are presented as Mean ± SEM. Statistical significance: * *p* < 0.05, *** *p* < 0.001, compared with WT control group; ^#^ *p* < 0.05, ^##^ *p* < 0.01, ^###^ *p* < 0.001, compared with WT H19Exo group; ^$$$^ *p* < 0.001, compared with WT H19Exo+M1 group; ^&^ *p* < 0.05, ^&&^ *p* < 0.01, ^&&&^ *p* < 0.001, compared with H19KO control group.

It is now well recognized that besides liver-resident macrophages, blood circulating BMDMs are also a vital source of hepatic macrophages and play a critical role in tissue repair and inflammatory responses [18,32]. BMDMs can transdifferentiate into either pro-inflammatory phenotype M1 or anti-inflammatory phenotype M2 macrophages in response to different stimuli, e.g., LPS and interferon-γ for M1 or IL-4 and IL-13 for M2 [15,33]. To determine the role of exosomal H19 in the activation and differentiation of BMDMs, we first examined the effect of H19Exo on M1 and M2 stimulator-induced polarization of WT and H19KO BMDMs. As shown in Figure 2A–D, H19Exo enhanced M1 stimulator-induced mRNA expression of M1 markers (IL-6, IL-1β, Cox-2, and CCL-5) in WT BMDMs, but not H19KO BMDMs. In addition, H19Exo increased M1 stimulator-induced mRNA levels of TNF-α and CXCL10 in both WT and H19KO BMDMs (Supplementary Figure S3A,B). Expression of macrophage polarization M2 markers, including CCL-24, CCL-17, IL-10, and Tgf-β, was not affected by H19Exo (data not shown). Surprisingly, the basal protein level of CCL-2 was almost undetectable in H19KO BMDMs when compared to that in WT BMDMs (Figure 2E). Although H19Exo did not further increase CCL-2 expression in WT BMDMs, it significantly increased CCR-2 protein expression in both WT and H19KO BMDM (Figure 2E). These results suggested that cholangiocyte-derived exosomal H19 played a critical role in the regulation of chemotaxis and BMDM infiltration into the liver.

### 3.3. Effects of Cholangiocyte-Released Exosomal H19 on BMDM Differentiation and Migration

**Figure 3:**
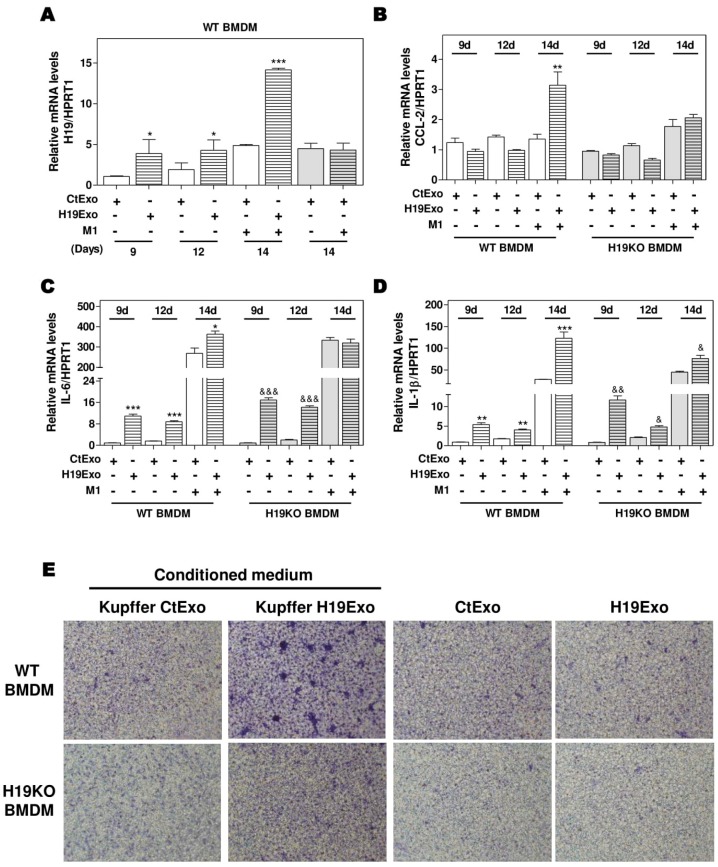
Cholangiocyte-derived exosomal H19 promotes BMDM differentiation and macrophage migration. (**A**–**D**) Mouse BMDM cells were isolated and cultured for 7 days. BMDMs were then changed with fresh differentiation medium with CtExo or H19Exo and collected on day 9 and 12. On day 14, BMDMs were treated with CtExo or H19Exo and M1 stimulators for another 24 h. The relative mRNA levels of H19, CCL-2, IL-6, and IL-1β were measured by real-time RT-PCR and normalized using HPRT1. (**E**) Kupffer cells in the transwell bottom chambers were treated with the conditioned medium from Kupffer cells treated with CtExo or H19Exo or directly treated with CtExo or H19Exo for 24 h. Images of migrating BMDMs on the top of the transwell chamber were captured, as described in Materials and Methods. Representative images of migration assay are shown. Results from at least three independent experiments are presented as Mean ± SEM. Statistical significance: * *p* < 0.05, ** *p* < 0.01, *** *p* < 0.001, compared with WT control group; ^&^ *p* < 0.05, ^&&^ *p* < 0.01, ^&&&^ *p* < 0.001, compared with H19KO control group.

Differentiation of BMDMs represents a crucial step in the progression of hepatic inflammation. To investigate the role of ExoH19 in BMDM differentiation, we cultured both WT and H19KO BMDMs for 7 days and then treated the cells with CtExo or H19Exo for another 7 days. As shown in Figure 3A, H19 expression was gradually increased during BMDM differentiation from day 9 to day 14. H19Exo from cholangiocytes further upregulated H19 mRNA levels in WT BMDMs during differentiation, when compared with CtExo treatment. The mRNA levels of H19 in H19KO BMDMs were also upregulated after 7-day H19Exo treatment, but much less than that in WT BMDMs (Supplementary Figure S4A). Interestingly, although CCL-2 expression was not statistically significant during BMDM differentiation, treatment of H19Exo from cholangiocytes significantly upregulated CCL-2 levels in WT BMDMs compared with that in H19KO BMDMs after 14 days treatment (Figure 3B). In line with these results, H19Exo upregulated the mRNA expression levels of M1 markers, including IL-6 and IL-1β, during BMDMs differentiation in both WT and H19KO (Figure 3C,D). Since H19Exo only promoted M1 stimulator-induced CCL-2 in WT BMDMs, we further used the transwell assay to examine the effect of H19Exo-induced activation of Kupffer cells on BMDM migration. As shown in Figure 3E (upper panel; Supplementary Figure S4B), the conditioned medium from H19Exo-treated Kupffer cells promoted the migration of WT BMDMs but was less effective in H19KO BMDMs. However, H19Exo alone had no effect on cell migration for both WT and H19KO BMDMs (Figure 3E, lower panel; Supplementary Figure S4B). These results suggested that cholangiocyte-derived exosomal H19 promoted WT BMDM cell migration via the activation of Kupffer cells.

### 3.4. Effects of CCL-2 on Exosomal H19-Induced Macrophage Activation and BMDM Migration

**Figure 4:**
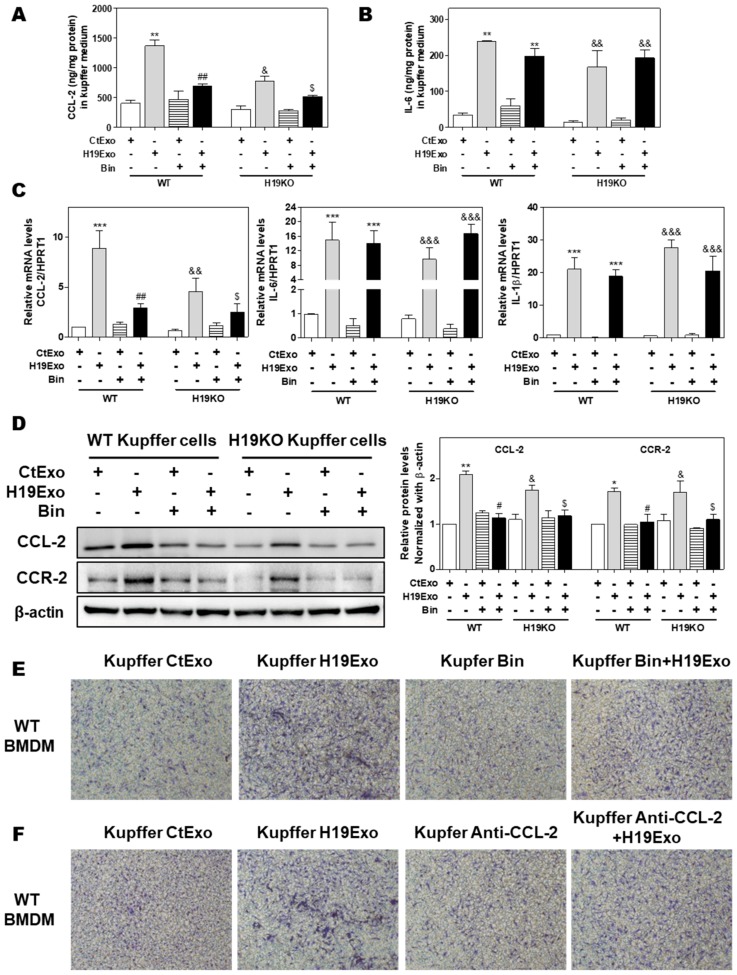
Effect of CCL-2 on exosomal H19-induced macrophage activation and migration. (**A–F**) Mouse Kupffer cells were treated with CtExo or H19Exo for 24 h, with or without pretreatment with Bindarit (Bin, 300 μM) and purified CCL-2 antibody (Anti-CCL-2, 20 μg/mL) for 2 h. (**A**,**B**) The levels of CCL-2 and IL-6 in the conditioned medium of Kupffer cells were measured by ELISA assay and normalized using protein concentration. (**C**) The relative mRNA levels of CCL-2, IL-6, and IL-1β were measured by real-time RT-PCR and normalized using HPRT1. (**D**) Representative immune blot images of CCL-2 and CCR-2 are shown. Relative protein levels were normalized using β-actin. (**E**,**F**) Representative images of migration assay are shown. Results from at least three independent experiments are presented as Mean ± SEM. Statistical significance: * *p* < 0.05, ** *p* < 0.01, *** *p* < 0.001, compared with WT control group; ^#^ *p* < 0.05, ^##^ *p* < 0.01, compared with WT H19Exo group; ^&^ *p* < 0.05, ^&&^ *p* < 0.01, ^&&&^ *p* < 0.001, compared with H19KO control group; ^$^ *p* < 0.05, compared with H19KO H19Exo group.

Previous studies showed that CCL-2-mediated chemotaxis was critical for driving monocytic infiltration and recruitment to livers via activation of CCR-2 during inflammatory processes [19]. Treatment of CCL-2 alone also resulted in increased expression of genes associated with classical M1 activation in BMDMs [34]. Given that the conditioned medium derived from H19Exo-treated Kupffer cells but not H19Exo itself contributed to BMDM migration (Figure 3E), chemokine secretion induced by H19Exo in Kupffer cells might play an essential role in promoting monocyte migration and recruitment. To test this hypothesis, we first examined the effect of a CCL-2 chemical inhibitor, Bindarit (Bin), on H19Exo-induced expression of pro-inflammatory cytokines and chemokines in Kupffer cells. As shown in Figure 4A,B, treatment with Bin significantly inhibited H19Exo-induced CCL-2 secretion from Kupffer cells but had no significant effect on IL-6 secretion. Similar results were observed in the real-time-PCR analysis (Figure 4C). Western Blot results demonstrated that H19KO Kupffer cells had lower basal expression levels of CCL-2 and CCR-2 when compared to WT Kupffer cells. Bin significantly downregulated H19Exo-induced protein expression of both CCL-2 and CCR-2 in Kupffer cells (Figure 4D). In support of these results, the transwell migration assay showed that both Bin and a CCL-2 neutralizing antibody significantly blocked the migration of WT BMDMs induced by the conditioned medium from the H19Exo-treated Kupffer cells (Figure 4E,F; Supplementary Figure S5). These results highlighted the vital role of Kupffer cell-released CCL-2 in H19Exo-induced inflammatory cell migration.

### 3.5. H19-Deficiency Ameliorates the Liver Cholestasis and Macrophage Activation in Both BDL and Mdr2^-/-^ Mice

**Figure 5:**
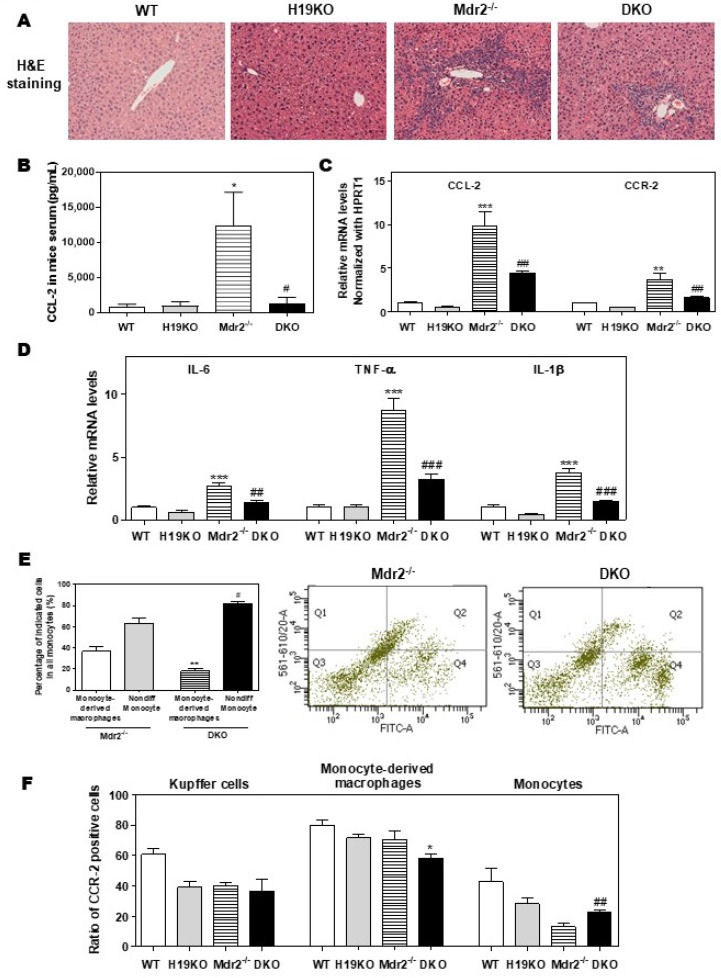
H19-enriched exosomes promote liver inflammation and macrophage activation in Mdr2^-/-^ mice. WT, H19KO, Mdr2^-/-^ mice, and DKO mice (both male and female at 100-day old) were sacrificed. (**A**) Representative images of H&E staining are shown. (**B**) CCL-2 levels in the serum. (**C**,**D**) The relative mRNA levels of hepatic CCL-2, CCR-2, IL-6, TNF-α, and IL-1β were determined by real-time RT-PCR and normalized using HPRT1. Statistical significance: * *p* < 0.05, ** *p* < 0.01, *** *p* < 0.001, compared with WT mice; ^#^ *p* < 0.05, ^##^ *p* < 0.01, ^###^ *p* < 0.001, compared with Mdr2^-/-^ mice. (**E**,**F**) Cell type-specific markers were used to determine the cell population using flow cytometry analysis. (**E**) Representative flow cytometry results and images of the percentage of indicated cells in all monocytes are shown. (**F**) The ratio of CCR-2 (C-C chemokine receptor 2) positive cells in Kupffer cells, monocyte-derived macrophages, and monocytes. Statistical significance: * *p* < 0.05, ** *p* < 0.01, compared with monocyte-derived macrophages in Mdr2^-/-^ mice; ^#^ *p* < 0.05, ^##^ *p* < 0.01, compared with non-differentiated monocytes in Mdr2^-/-^ mice (*n* > 6).

To further define the crucial role of H19 in macrophage activation and differentiation, we generated DKO mice with a C57BL/6J background, as described previously [9]. As shown in Figure 5A, H19 deficiency in DKO mice significantly reduced inflammatory cell infiltration and liver fibrosis when compared with Mdr2^-/-^ mice. Consistently, Mdr2 deficiency-induced upregulation of serum CCL-2 level was blocked in DKO mice (Figure 5B). The hepatic mRNA levels of CCL-2, CCR-2, and activated M1 marker genes, including IL-6, TNF-α, IL-1β, CD11b, CXCL10, and CD86, were all significantly downregulated in DKO mice (Figure 5C,D; Supplementary Figure S6A–C). To further characterize the role of H19 in macrophage activation in cholestatic liver injury, flow cytometry was used to separate resident Kupffer cells, monocytes, and monocyte-derived macrophages and identify the CCR-2 positive cell population. The CD45^+^ cells were further stained with F4/80 and CD11b antibodies to differentiate Kupffer cells (F4/80^+^-CD11b^-^), monocytes (F4/80^−^-CD11b^+^), and monocyte-derived macrophages (F4/80^+^-CD11b^+^). The gating strategy for flow cytometry analysis is shown in Supplementary Figure S7. As shown in Figure 5E, significant differentiation arrest of the monocyte population in DKO mice was observed. Specifically, about 36.7% of monocytes (Q1 and Q2) were differentiated into macrophages (Q2) in the liver of Mdr2^-/-^ mice. However, only 18.2% of monocytes in the liver of H19KO mice differentiated into macrophages (Supplementary Figure S8A). Interestingly, CCR-2 positive monocyte-derived macrophages were decreased, but on the other hand, CCR-2 positive monocytes were increased in DKO mice, when compared to Mdr2^-/-^ mice (Figure 5F; Supplementary Figure S8B).

**Figure 6:**
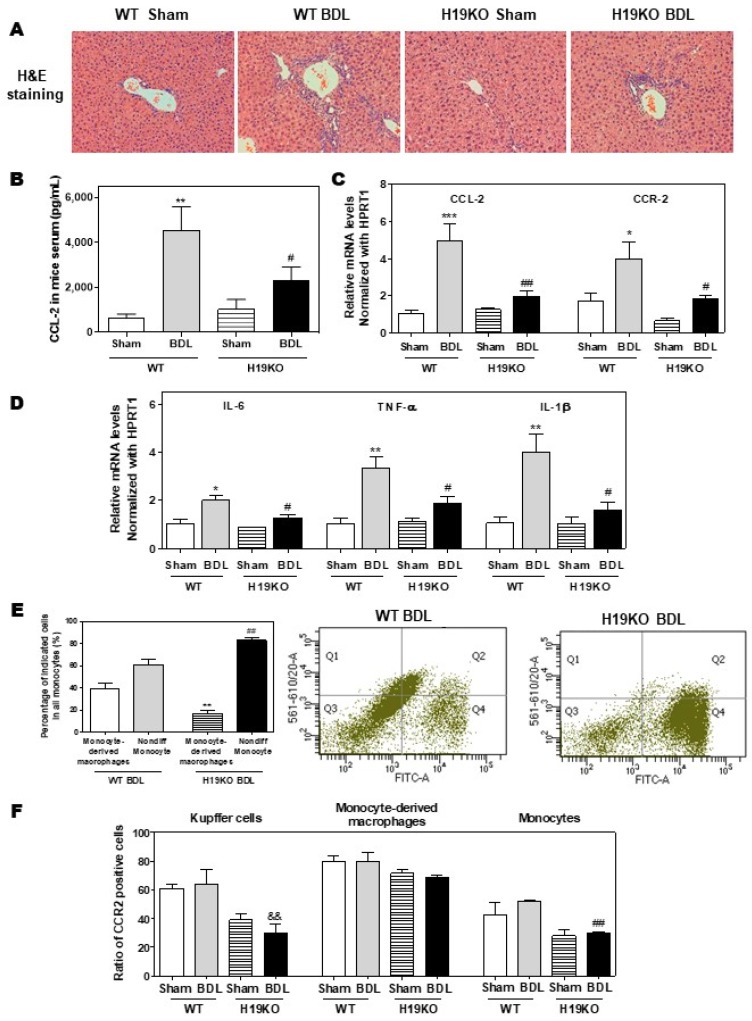
H19-enriched exosomes promote liver inflammation and macrophage activation in bile duct ligation (BDL) mice. Both WT and H19KO mice (both male and female at 12 weeks old) were subjected to sham operation or BDL for 2 weeks. (**A**) Representative images of H&E staining are shown. (**B**) CCL-2 levels in the serum. (**C**,**D**) The relative mRNA levels of hepatic CCL-2, CCR-2, IL-6, TNF-α, and IL-1β were determined by real-time RT-PCR and normalized using HPRT1. Statistical significance: * *p* < 0.05, ** *p* < 0.01, *** *p* < 0.001, compared with WT sham mice; ^#^ *p* < 0.05, ^##^ *p* < 0.01, compared with WT BDL mice (*n* > 6). (**E**,**F**) Cell type-specific markers were used to determine the cell population using flow cytometry analysis. (**E**) Representative flow cytometry results and images of the percentage of indicated cells in all monocytes are shown. (**F**) The ratio of CCR-2 positive cells in Kupffer cells, monocyte-derived macrophages, and monocytes. Statistical significance: ** *p* < 0.01, compared with monocyte-derived macrophages in WT BDL mice; ^##^ *p* < 0.01, compared with non-differentiated monocytes in WT BDL mice; ^&&^ *p* < 0.01, compared with Kupffer cells in WT BDL mice (*n* > 6).

Additionally, in a 2-week BDL mouse model, H19KO mice had a less cholestatic liver injury, significant reduction of inflammatory cell infiltration, and bile duct proliferation, as shown in Figure 6A. Similar to the findings in Mdr2^-/-^ mice, BDL significantly increased serum CCL-2 levels in WT mice but had less effect in H19KO mice (Figure 6B). Real-time PCR analysis also showed that the BDL significantly upregulated the mRNA levels of CCL-2, CCR-2, and M1 activation marker genes, including IL-6, TNF-α, and IL-1β, in WT BDL mice, but had much less impact on H19KO mice (Figure 6C,D). In addition, H19 deficiency also significantly prevented the differentiation of monocytes to macrophages in the BDL mouse model (Figure 6E). The ratio of differentiated monocytes to all monocytes was about 38.9% in WT BDL mice, whereas only 17.2% in H19KO BDL mice (Figure 6E; Supplementary Figure S9A). Furthermore, the number of CCR-2 positive Kupffer cells and undifferentiated monocytes was significantly reduced in H19KO BDL mice compared to that in WT BDL mice (Figure 6F; Supplementary Figure S9B). These results suggested that H19 deficiency in vivo significantly inhibited the differentiation of monocytes to macrophages and thus alleviated inflammatory responses in both Mdr2^-/-^ mice and BDL mice.

### 3.6. Aberrant Expression of CCL-2 and IL-6 in PBC and PSC Patients

**Figure 7:**
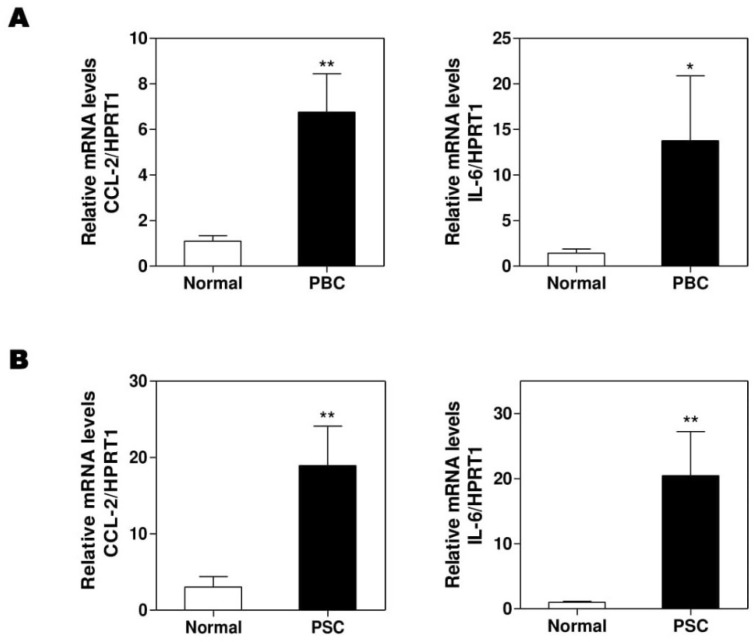
The expressions of CCL-2 and IL-6 in normal, primary biliary cholangitis (PBC), and primary sclerosing cholangitis (PSC) human livers. Total RNAs were isolated from frozen liver tissues of normal control or PBC and PSC patients. The mRNA levels of CCL-2 and IL-6 were determined by real-time RT-PCR and normalized using HPRT1. (**A**), PBC; (**B**). PSC. Statistical significance: * *p* < 0.05, ** *p* < 0.01, compared with normal human (*n* = 8).

To further confirm that our findings in Mdr2^-/-^ and BDL mice are translatable to human cholestatic liver diseases, we obtained several frozen liver tissues of the sex-matched PBC, PSC patients, and normal controls from the National Institutes of Health-sponsored Liver Tissue Cell Distribution System. We previously reported that hepatic H19 mRNA levels were significantly increased in PSC and PBC patients [14]. As expected, the real-time PCR results showed that the hepatic mRNA levels of CCL-2 and IL-6 were increased in both PBC (Figure 7A) and PSC (Figure 7B) patients when compared to healthy control.

## 4. Discussion

**Figure 8:**
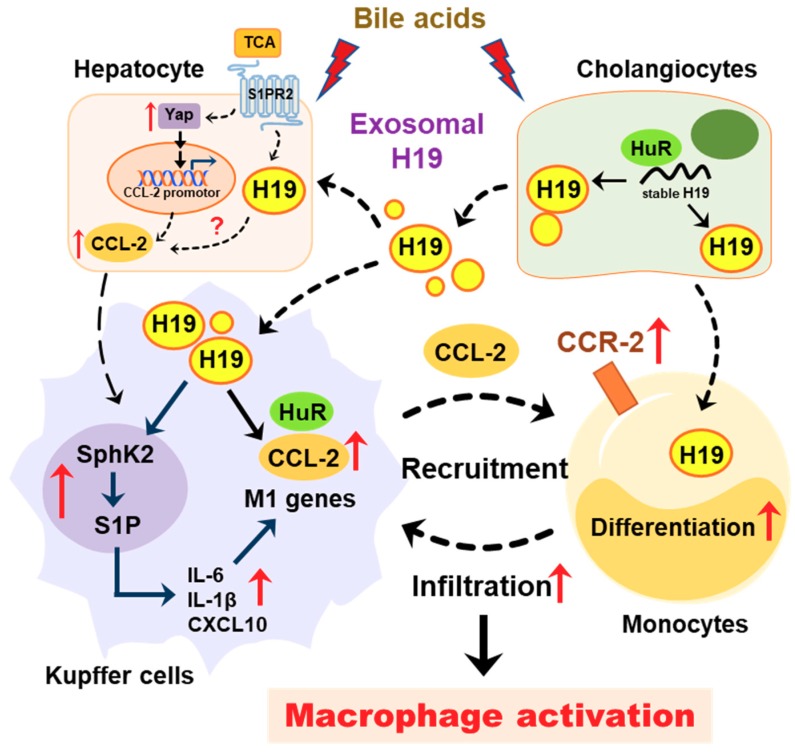
Schematic diagram of the proposed mechanism by which cholangiocyte-derived H19 exosomes promote hepatic inflammation and macrophage activation during cholestatic liver injury.

Cholangiocytes are the primary targets in the pathogenesis of cholestatic liver diseases. Biliary obstruction and abnormal bile formation result in the accumulation of bile acids, which causes progressive bile duct destruction and hepatic inflammation, and further contributes to fibrosis, cirrhosis, and eventually liver failure [35,36,37]. It has been well documented that hepatic inflammation is an essential driving force for liver fibrosis by recruiting inflammatory cells and activating HSCs [33,38]. Pro-inflammatory cytokines repress the expression and function of hepatocellular transporters and further result in cholestatic hepatitis [35]. Our previous study reported that bile acid-induced upregulation of H19 in cholangiocytes contributed to the dysregulation of hepatic bile acids and the progression of liver fibrosis in both Mdr2^-/-^ mice and BDL mice. We recently showed that cholangiocyte-derived H19Exo not only disturbed the bile acid homeostasis through suppressing SHP expression in hepatocytes but also directly promoted the activation and proliferation of HSCs under cholestatic conditions [9,14]. However, it remains unclear whether and how cholangiocyte-derived H19Exo regulates macrophage infiltration, differentiation, and activation. The current study showed that cholangiocyte-derived H19Exo played an essential role in hepatic inflammation and cholestatic liver disease progression (Figure 8).

In addition to direct cell-to-cell contact, EVs have recently drawn considerable attention as critical mediators of intercellular communication in normal physiological homeostasis and pathogenesis of diseases [39]. Exosomes are small EVs released by many different types of cells, including hepatocytes, cholangiocytes, macrophages, stem cells, and tumor cells, and can transfer multiple cargos, including DNA, miRNA, lncRNA, mRNA, protein, and lipids, to adjacent tissues or distant organs through circulation [40,41,42,43,44]. Numerous studies suggest that exosomal cargos in various biofluids can be used as a promising diagnostic biomarker for assessing various human liver diseases, including hepatitis, drug-induced liver injury, non-alcoholic and alcoholic fatty liver diseases, hepatocellular carcinoma, and cholangiocarcinoma [30,45]. Emerging evidence further indicates that hepatic cell-derived exosomes and their cargo play essential roles in the regulation of hepatic cell proliferation and differentiation and modulation of inflammatory responses. Masyuk et al. reported that biliary exosomes influenced cholangiocyte proliferation by regulating the miRNA-15A expression and intracellular signaling pathways [46]. It has also been reported that biliary exosomes enhance the proliferation of CD4^+^ and CD8^+^ T cells and monocytes in the liver during inflammation [47]. Previous studies also suggested that, in response to alcohol or lipid exposure, hepatocyte-derived EVs stimulated expression of inflammatory cytokines and chemokines, including IL6, IL-1β, and CXCL10, and induced activation and chemotaxis of Kupffer cells and mouse BMDMs [48,49,50]. A recent study further demonstrated that inflamed endothelial cell-derived EVs mediated selective transfer of inflammatory chemokines and cytokines to target monocytes and reprogramed them toward M1 or M2 phenotypes [51]. Additionally, it has been shown that exosomes derived from alcoholic hepatocytes transferred miRNA-122 to macrophages, increased expression of pro-inflammatory cytokines, and sensitized monocytes to LPS stimulation [52]. Furthermore, mesenchymal stem cell-derived exosomal miR-17 reduced inflammatory factor secretion by suppressing inflammasome activation in hepatic macrophages [53]. However, the role of cholangiocyte-derived exosomes in the regulation of hepatic macrophages remains largely unknown.

Here, we reported that exosomes are essential for intercellular communication among various hepatic cells, including cholangiocytes, Kupffer cells, and monocyte-derived macrophages, and play critical roles in the pathogenesis of cholestatic liver injury. LncRNA H19, a paternally imprinted and maternally expressed gene, is repressed after birth, but highly expressed in malignancies [54]. Our recent study showed that H19 was mainly expressed in cholangiocytes, and aberrant expression of H19 was associated with the disturbance of bile acids and hepatic inflammation in different cholestatic mouse models [9,14]. H19 containing cholangiocyte-derived exosomes were rapidly taken up by HSCs, Kupffer cells, and hepatocytes [9]. A previous study reported that overexpression of H19 increased vascular inflammation by increasing the expression of IL-6 and CCL-2 and promoting macrophage infiltration [55]. In the present study, we reported that cholangiocyte-derived H19Exo increased the expression and secretion of CCL-2 in WT Kupffer cells, but had less effect on H19KO Kupffer cells (Figure 1). We further showed that H19Exo not only promoted M1 polarization and migration of WT BMDMs but also gradually increased the release of pro-inflammatory cytokines and promoted differentiation of monocyte-derived macrophages (Figure 2 and Figure 3). Interestingly, the basal level of CCL-2 in H19KO BMDMs was almost undetectable compared with that in WT BMDMs (Figure 2E), indicating a critical role for H19 in the regulation of chemokine expression. In cultured primary Kupffer cells, H19Exo-induced CCL-2/CCR-2 expression and BMDM migration were significantly reduced by the CCL-2 inhibitor Bindarit or neutralization of CCL-2 using a monoclonal antibody (Figure 4). These results were consistent with a previous study, which suggested that knockout of CCL-2 significantly mitigated hepatic neutrophil chemotaxis and inflammation in cholic acid feeding- and 7-day BDL-induced cholestatic mice [24]. In support of the in vitro studies, we identified that H19 deficiency significantly downregulated CCR-2 positive cells and prevented the differentiation from monocytes to monocyte-derived macrophages in both Mdr2^-/-^ and BDL cholestatic mouse models (Figure 5 and Figure 6). These findings suggested that H19-carrying exosomes are important players in the chemokine-mediated inflammatory cell infiltration and progression of hepatic inflammation.

Sphingosine kinase 2 (SphK2) is highly expressed in the liver and phosphorylates sphingosine to generate sphingosine-1-phosphate (S1P) in the nucleus [56,57]. Recently, we found that the hepatic H19 mRNA level was positively correlated with the hepatic SphK2 mRNA level [8]. The role of SphK2 in regulating H19-mediated inflammatory response remains to be fully studied.

In conclusion, as illustrated in Figure 8, under cholestatic conditions, accumulated bile acids increased lncRNA H19 levels in cholangiocytes and cholangiocyte-derived exosome cargos. H19Exo could be uptaken by Kupffer cells and monocytes, which further promoted differentiation of monocyte-derived macrophages, macrophage activation, and inflammatory cell migration, and thus promoted hepatic inflammation during cholestasis. This study suggested that targeting cholangiocyte-derived exosomal H19 is a potential therapeutic strategy for the treatment of cholestatic liver diseases by modulation of hepatic inflammation.

## Supplementary Materials

The following are available online at https://www.mdpi.com/2073-4409/9/1/190/s1, Figure S1: Effects of exosomal H19 on CCR-2 expression in Kupffer cells; Figure S2: Effects of exosomal H19 and LPS on Kupffer cell activation; Figure S3: Effects of exosomal H19 on TNF-α and CXCL10 expression in BMDMs; Figure S4: Effects of exosomal H19 on H19 expression and cell migration in BMDMs; Figure S5: Effect of Bindarit and anti-CCL-2 antibody on exosomal H19-induced macrophage migration; Figure S6: Hepatic mRNA levels of inflammatory factors in Mdr2^-/-^ mice; Figure S7: Gating strategy for the identification of hepatic cells by FACS; Figure S8: Flow cytometry analysis of Mdr2^-/-^ mice; Figure S9: Flow cytometry analysis of BDL mice; Table S1: List of antibodies

## Abbreviations

BDL, bile duct ligation; BMDM, bone marrow-derived macrophage; CCL-2, chemokine (C-C motif) ligand 2; CXCL10, chemokine (C-X-C motif) ligand 10; DKO mice, Mdr2^-/-^H19^maternalΔExon1/+^ mice; ELISA, Enzyme-linked Immunosorbent Assay; EV, extracellular vesicles; H19KO mice, H19^maternalΔExon1/+^ mice; HSC, hepatic stellate cells; IL-6, interleukin 6; lncRNA, long non-coding RNA; Mdr2, multidrug resistance 2; PBC, primary biliary cholangitis; PSC, primary sclerosing cholangitis; S1PR2, sphingosine-1 phosphate receptor 2; S1PR2, sphingosine 1-phosphate receptor 2; SHP, short heterodimer partner; TCA, taurocholate acid; WT, wild type.

## References

[1] Lazaridis K.N., LaRusso N.F. (2015). The Cholangiopathies. Mayo. Clin. Proc..

[2] O’Hara S.P., Tabibian J.H., Splinter P.L., LaRusso N.F. (2013). The dynamic biliary epithelia: Molecules, pathways, and disease. J. Hepatol..

[3] Gidwaney N.G., Pawa S., Das K.M. (2017). Pathogenesis and clinical spectrum of primary sclerosing cholangitis. World J. Gastroenterol..

[4] Banales J.M., Huebert R.C., Karlsen T., Strazzabosco M., LaRusso N.F., Gores G.J. (2019). Cholangiocyte pathobiology. Nat. Rev. Gastroenterol. Hepatol..

[5] Wang Y., Aoki H., Yang J., Peng K., Liu R., Li X., Qiang X., Sun L., Gurley E.C., Lai G. (2017). The role of sphingosine 1-phosphate receptor 2 in bile-acid-induced cholangiocyte proliferation and cholestasis-induced liver injury in mice. Hepatology.

[6] Liu R., Li X., Qiang X., Luo L., Hylemon P.B., Jiang Z., Zhang L., Zhou H. (2015). Taurocholate Induces Cyclooxygenase-2 Expression via the Sphingosine 1-phosphate Receptor 2 in a Human Cholangiocarcinoma Cell Line. J. Biol. Chem..

[7] Gabory A., Ripoche M.A., Le Digarcher A., Watrin F., Ziyyat A., Forne T., Jammes H., Ainscough J.F., Surani M.A., Journot L. (2009). H19 acts as a trans regulator of the imprinted gene network controlling growth in mice. Development.

[8] Xiao Y., Liu R., Li X., Gurley E.C., Hylemon P.B., Lu Y., Zhou H., Cai W. (2019). Long non-coding RNA H19 contributes to cholangiocyte proliferation and cholestatic liver fibrosis in biliary atresia. Hepatology.

[9] Liu R., Li X., Zhu W., Wang Y., Zhao D., Wang X., Gurley E.C., Liang G., Chen W., Lai G. (2019). Cholangiocyte-Derived Exosomal Long Noncoding RNA H19 Promotes Hepatic Stellate Cell Activation and Cholestatic Liver Fibrosis. Hepatology.

[10] Li X., Liu R., Yang J., Sun L., Zhang L., Jiang Z., Puri P., Gurley E.C., Lai G., Tang Y. (2017). The role of long noncoding RNA H19 in gender disparity of cholestatic liver injury in multidrug resistance 2 gene knockout mice. Hepatology.

[11] He C., Zheng S., Luo Y., Wang B. (2018). Exosome Theranostics: Biology and Translational Medicine. Theranostics..

[12] Martinez M.C., Andriantsitohaina R. (2017). Extracellular Vesicles in Metabolic Syndrome. Circ. Res..

[13] Sato K., Kennedy L., Liangpunsakul S., Kusumanchi P., Yang Z., Meng F., Glaser S., Francis H., Alpini G. (2019). Intercellular Communication between Hepatic Cells in Liver Diseases. Int. J. Mol. Sci..

[14] Li X., Liu R., Huang Z., Gurley E.C., Wang X., Wang J., He H., Yang H., Lai G., Zhang L. (2018). Cholangiocyte-derived exosomal long noncoding RNA H19 promotes cholestatic liver injury in mouse and humans. Hepatology.

[15] Murray P.J., Wynn T.A. (2011). Protective and pathogenic functions of macrophage subsets. Nat. Rev. Immunol..

[16] Klein I., Cornejo J.C., Polakos N.K., John B., Wuensch S.A., Topham D.J., Pierce R.H., Crispe I.N. (2007). Kupffer cell heterogeneity: Functional properties of bone marrow derived and sessile hepatic macrophages. Blood.

[17] Li X., Yao W., Yuan Y., Chen P., Li B., Li J., Chu R., Song H., Xie D., Jiang X. (2017). Targeting of tumour-infiltrating macrophages via CCL2/CCR2 signalling as a therapeutic strategy against hepatocellular carcinoma. Gut.

[18] Tacke F., Zimmermann H.W. (2014). Macrophage heterogeneity in liver injury and fibrosis. J. Hepatol..

[19] Serbina N.V., Jia T., Hohl T.M., Pamer E.G. (2008). Monocyte-mediated defense against microbial pathogens. Annu. Rev. Immunol..

[20] Baeck C., Wehr A., Karlmark K.R., Heymann F., Vucur M., Gassler N., Huss S., Klussmann S., Eulberg D., Luedde T. (2012). Pharmacological inhibition of the chemokine CCL2 (MCP-1) diminishes liver macrophage infiltration and steatohepatitis in chronic hepatic injury. Gut.

[21] Guicciardi M.E., Trussoni C.E., Krishnan A., Bronk S.F., Lorenzo Pisarello M.J., O’Hara S.P., Splinter P.L., Gao Y., Vig P., Revzin A. (2018). Macrophages contribute to the pathogenesis of sclerosing cholangitis in mice. J. Hepatol..

[22] Seki E., de Minicis S., Inokuchi S., Taura K., Miyai K., van Rooijen N., Schwabe R.F., Brenner D.A. (2009). CCR2 promotes hepatic fibrosis in mice. Hepatology.

[23] Mitchell C., Couton D., Couty J.P., Anson M., Crain A.M., Bizet V., Renia L., Pol S., Mallet V., Gilgenkrantz H. (2009). Dual role of CCR2 in the constitution and the resolution of liver fibrosis in mice. Am. J. Pathol..

[24] Cai S.Y., Ouyang X., Chen Y., Soroka C.J., Wang J., Mennone A., Wang Y., Mehal W.Z., Jain D., Boyer J.L. (2017). Bile acids initiate cholestatic liver injury by triggering a hepatocyte-specific inflammatory response. J.C.I. Insight.

[25] Zhang X., Cao R., Liu R., Zhao R., Huang Y., Gurley E.C., Hylemon P.B., Pandak W.M., Wang G., Zhang L. (2014). Reduction of the HIV protease inhibitor-induced ER stress and inflammatory response by raltegravir in macrophages. PLoS ONE.

[26] Li X., Liu R., Luo L., Yu L., Chen X., Sun L., Wang T., Hylemon P.B., Zhou H., Jiang Z. (2017). Role of AMP-activated protein kinase alpha1 in 17alpha-ethinylestradiol-induced cholestasis in rats. Arch. Toxicol..

[27] Liu R., Zhao R., Zhou X., Liang X., Campbell D.J., Zhang X., Zhang L., Shi R., Wang G., Pandak W.M. (2014). Conjugated bile acids promote cholangiocarcinoma cell invasive growth through activation of sphingosine 1-phosphate receptor 2. Hepatology.

[28] Mills C.D., Kincaid K., Alt J.M., Heilman M.J., Hill A.M. (2000). M-1/M-2 macrophages and the Th1/Th2 paradigm. J. Immunol..

[29] Martinez F.O., Gordon S. (2014). The M1 and M2 paradigm of macrophage activation: Time for reassessment. F1000Prime Rep..

[30] Eguchi A., Kostallari E., Feldstein A.E., Shah V.H. (2019). Extracellular vesicles, the liquid biopsy of the future. J. Hepatol..

[31] Han W., Duan Z. (2019). Roles of exosomes in liver metastases: Novel diagnosis and treatment choices. J. Cell. Physiol..

[32] Ju C., Tacke F. (2016). Hepatic macrophages in homeostasis and liver diseases: From pathogenesis to novel therapeutic strategies. Cell. Mol. Immunol..

[33] Zhou D., Yang K., Chen L., Wang Y., Zhang W., Xu Z., Zuo J., Jiang H., Luan J. (2017). Macrophage polarization and function: New prospects for fibrotic disease. Immunol. Cell. Biol..

[34] Carson W.F.t., Salter-Green S.E., Scola M.M., Joshi A., Gallagher K.A., Kunkel S.L. (2017). Enhancement of macrophage inflammatory responses by CCL2 is correlated with increased miR-9 expression and downregulation of the ERK1/2 phosphatase Dusp6. Cell. Immunol..

[35] Zollner G., Trauner M. (2008). Mechanisms of cholestasis. Clin Liver Dis.

[36] Glaser S.S., Gaudio E., Miller T., Alvaro D., Alpini G. (2009). Cholangiocyte proliferation and liver fibrosis. Expert Rev. Mol. Med..

[37] Sato K., Meng F., Giang T., Glaser S., Alpini G. (2018). Mechanisms of cholangiocyte responses to injury. Biochim Biophys. Acta Mol. Basis Dis..

[38] Mack M. (2018). Inflammation and fibrosis. Matrix Biol.

[39] Xie F., Feng S., Yang H., Mao Y. (2019). Extracellular vesicles in hepatocellular cancer and cholangiocarcinoma. Ann. Transl. Med..

[40] McDaniel K., Wu N., Zhou T., Huang L., Sato K., Venter J., Ceci L., Chen D., Ramos-Lorenzo S., Invernizzi P. (2019). Amelioration of Ductular Reaction by Stem Cell Derived Extracellular Vesicles in MDR2 Knockout Mice via Lethal-7 microRNA. Hepatology.

[41] Chen L., Xiang B., Wang X., Xiang C. (2017). Exosomes derived from human menstrual blood-derived stem cells alleviate fulminant hepatic failure. Stem Cell Res. Ther..

[42] Nojima H., Freeman C.M., Schuster R.M., Japtok L., Kleuser B., Edwards M.J., Gulbins E., Lentsch A.B. (2016). Hepatocyte exosomes mediate liver repair and regeneration via sphingosine-1-phosphate. J. Hepatol..

[43] Zhang H., Deng T., Liu R., Bai M., Zhou L., Wang X., Li S., Wang X., Yang H., Li J. (2017). Exosome-delivered EGFR regulates liver microenvironment to promote gastric cancer liver metastasis. Nat. Commun..

[44] Sasaki R., Kanda T., Yokosuka O., Kato N., Matsuoka S., Moriyama M. (2019). Exosomes and Hepatocellular Carcinoma: From Bench to Bedside. Int. J. Mol. Sci..

[45] Cho Y.E., Song B.J., Akbar M., Baek M.C. (2018). Extracellular vesicles as potential biomarkers for alcohol- and drug-induced liver injury and their therapeutic applications. Pharmacol. Ther..

[46] Masyuk A.I., Huang B.Q., Ward C.J., Gradilone S.A., Banales J.M., Masyuk T.V., Radtke B., Splinter P.L., LaRusso N.F. (2010). Biliary exosomes influence cholangiocyte regulatory mechanisms and proliferation through interaction with primary cilia. Am. J Physiol. Gastrointest. Liver Physiol..

[47] Wang Y., Wang G., Wang Z., Zhang H., Zhang L., Cheng Z. (2014). Chicken biliary exosomes enhance CD4(+)T proliferation and inhibit ALV-J replication in liver. Biochem. Cell Biol..

[48] Verma V.K., Li H., Wang R., Hirsova P., Mushref M., Liu Y., Cao S., Contreras P.C., Malhi H., Kamath P.S. (2016). Alcohol stimulates macrophage activation through caspase-dependent hepatocyte derived release of CD40L containing extracellular vesicles. J. Hepatol..

[49] Hirsova P., Ibrahim S.H., Krishnan A., Verma V.K., Bronk S.F., Werneburg N.W., Charlton M.R., Shah V.H., Malhi H., Gores G.J. (2016). Lipid-Induced Signaling Causes Release of Inflammatory Extracellular Vesicles From Hepatocytes. Gastroenterology.

[50] Ibrahim S.H., Hirsova P., Tomita K., Bronk S.F., Werneburg N.W., Harrison S.A., Goodfellow V.S., Malhi H., Gores G.J. (2016). Mixed lineage kinase 3 mediates release of C-X-C motif ligand 10-bearing chemotactic extracellular vesicles from lipotoxic hepatocytes. Hepatology.

[51] Hosseinkhani B., Kuypers S., van den Akker N.M.S., Molin D.G.M., Michiels L. (2018). Extracellular Vesicles Work as a Functional Inflammatory Mediator Between Vascular Endothelial Cells and Immune Cells. Front. Immunol..

[52] Momen-Heravi F., Bala S., Kodys K., Szabo G. (2015). Exosomes derived from alcohol-treated hepatocytes horizontally transfer liver specific miRNA-122 and sensitize monocytes to LPS. Sci. Rep..

[53] Liu Y., Lou G., Li A., Zhang T., Qi J., Ye D., Zheng M., Chen Z. (2018). AMSC-derived exosomes alleviate lipopolysaccharide/d-galactosamine-induced acute liver failure by miR-17-mediated reduction of TXNIP/NLRP3 inflammasome activation in macrophages. EBioMedicine.

[54] Pope C., Mishra S., Russell J., Zhou Q., Zhong X.B. (2017). Targeting H19, an Imprinted Long Non-Coding RNA, in Hepatic Functions and Liver Diseases. Diseases.

[55] Sun Y., Zhong L., He X., Wang S., Lai Y., Wu W., Song H., Chen Y., Yang Y., Liao W. (2019). LncRNA H19 promotes vascular inflammation and abdominal aortic aneurysm formation by functioning as a competing endogenous RNA. J. Mol. Cell. Cardiol..

[56] Leclercq T.M., Pitson S.M. (2006). Cellular signalling by sphingosine kinase and sphingosine 1-phosphate. IUBMB Life.

[57] Kwong E.K., Liu R., Zhao D., Li X., Zhu W., Wang X., Gurley E.C., Lai G., Liu J., Hylemon P.B. (2019). The role of sphingosine kinase 2 in alcoholic liver disease. Dig. Liver. Dis..

