# Supplementary material for: Cholangiocyte-Derived Exosomal lncRNA H19 Promotes Macrophage Activation and Hepatic Inflammation under Cholestatic Conditions
Source: Cells. 2020 Jan 11;9(1):190. doi: 10.3390/cells9010190 (PMC7016679; doi:10.3390/cells9010190)

Supplementary figure legends

Supplementary figure 1. Effects of exosomal H19 on CCR-2 expression in Kupffer cells. Mouse Kupffer cells were treated with control MLE-derived exosomes (CtExo) or H19-overexpressing MLE-derived exosomes (H19Exo) for 24 h. The relative mRNA level of CCR-2 was measured by real-time RT-PCR and normalized using HPRT1. Results from at least 3 independent experiments are presented as Mean ± SEM. Statistical significance: ****P*<0.001, compared with WT control group; $^{\&}$*P*<0.05, compared with the H19KO control group.

Supplementary figure 2. Effects of exosomal H19 and LPS on Kupffer cell activation. Mouse Kupffer cells were treated with CtExo, H19Exo with or without LPS (10 ng/ml) for 24 h. **(A)** The levels of TNF-α in the conditioned medium of Kupffer cells were measured by ELISA assay and normalized using protein concentration. **(B-D)** The relative mRNA levels of IL12p40, CXCL10 and CCL-5 were measured by real-time RT-PCR and normalized using HPRT1. Results from at least 3 independent experiments are presented as Mean ± SEM. Statistical significance: ***P*<0.01, ****P*<0.001, compared with WT control group; $^{\#}$*P*<0.05, compared with WT H19Exo group; $^{\&}$*P*<0.05, $^{\&\&}$*P*<0.01, compared with H19KO control group.

Supplementary figure 3. Effects of exosomal H19 on TNF-α and CXCL10 expression in BMDMs. Mouse BMDM cells were treated with CtExo, H19Exo, M1 (LPS, 10 ng/ml and IFN-γ, 100 ng/ml) or M2 stimulators (IL-4, 20 ng/ml and IL-13, 20 ng/ml) for 24 h. The relative mRNA level of TNF-α **(A)** and CXCL10 **(B)** was measured by real-time RT-PCR and normalized using HPRT1. Results from at least

3 independent experiments are presented as Mean ± SEM. Statistical significance: ****P*<0.001, compared with WT control group; $^{\&\&\&}$*P*<0.001, compared with H19KO control group; $^{\$\$}$*P*<0.01, compared with WT H19Exo group.

Supplementary figure 4. Effects of exosomal H19 on H19 expression and cell migration in BMDMs. WT and H19KO BMDM cells were isolated and cultured for 7 days. BMDMs were then changed fresh differentiation medium with CtExo or H19Exo and collected on day 9 and 12. On day 14, BMDMs were treated with CtExo or H19Exo and M1 stimulators for 24 h. **(A)** The relative mRNA levels of H19 in H19KO BMDM were measured by real-time RT-PCR and normalized using HPRT1. **(B)** Relative migration area. Results from at least 3 independent experiments are presented as Mean ± SEM. Statistical significance: **P*<0.05, ***P*<0.01, ****P*<0.001, compared with WT control group.

Supplementary figure 5. Effect of Bindarit and anti-CCL-2 antibody on exosomal H19-induced macrophage migration. Mouse Kupffer cells were treated with CtExo or H19Exo for 24 h, with or without pretreatment of Bindarit (Bin, 300 μM) **(A)** and purified CCL-2 antibody (20 μg/ml) **(B)** for 2 h. **(A-B)** The relative migration area is shown. Results from at least 3 independent experiments are presented as Mean ± SEM. Statistical significance: **P*<0.05, compared with the control group; $^{\#}$*P*<0.05, compared with H19Exo group.

Supplementary figure 6. Hepatic mRNA levels of inflammatory factors in Mdr2$^{-/-}$ mice. WT, H19KO, Mdr2$^{-/-}$ mice, and DKO mice (both male and female at 100-day old) were sacrificed. **(A-C)** The relative mRNA levels of CD11b, CXCL10 and CD86,

were determined by real-time RT-PCR and normalized using HPRT1 as an internal control. Results from at least 3 independent experiments are presented as Mean ± SEM. Statistical significance: ***P<0.001, compared with WT mice; $^{\#}P$<0.05, $^{\#\#}P$<0.01, $^{\#\#\#}P$<0.001, compared with Mdr2$^{-/-}$ mice.

Supplementary figure 7. Gating strategy for the identification of hepatic cells by FACS. Negative control for the detection of CD45 (APC-Cy7), F4/80 (561-610), CD11b (FITC-A) and CCR-2 (Alexa 647-A). Representative graphs are shown.

Supplementary figure 8. Flow cytometry analysis of Mdr2$^{-/-}$ mice. WT, H19KO, Mdr2$^{-/-}$ mice and DKO mice (both male and female at 100-day old) were sacrificed. **(A, B)** Representative flow cytometry images of the percentage of indicated cells in all monocytes are shown (n>6).

Supplementary figure 9. Flow cytometry analysis of BDL mice. Both WT and H19KO mice (both male and female at 12 weeks old) were subjected to sham operation or BDL for 2 weeks. **(A, B)** Representative flow cytometry images of the percentage of indicated cells in all monocytes are shown (n>6).

Supplementary Table 1. List of antibodies

Antibody	Species	Source	Catalog #	Application/ dilution
CCL-2	Mouse	Santa Cruz	MA5-17040	WB (1:1000)
CCR-2	Rabbit	Abcam	ab203128	WB (1:1000)
CD45-APC/Cy7	Mouse	Biolegend	103116	Flow cytometry ($0.2\mu g/10^6$ cells)
CD16/CD32	Mouse	BD Biosciences	553142	Flow cytometry ($1\mu g/10^6$ cells)
F480-Alexa Fluor 594	Mouse	Biolegend	123140	Flow cytometry ($0.2\mu g/10^6$ cells)
CD11b-FITC	Mouse	Biolegend	101206	Flow cytometry ($0.2\mu g/10^6$ cells)
CD192 (CCR2)-Alexa Fluor 647	Mouse	Biolegend	150604	Flow cytometry ($0.2\mu g/10^6$ cells)
β-actin (JLA20)	Mouse	DSHB University of Iowa	JLA20	WB (1:500)

Supplementary figure 1

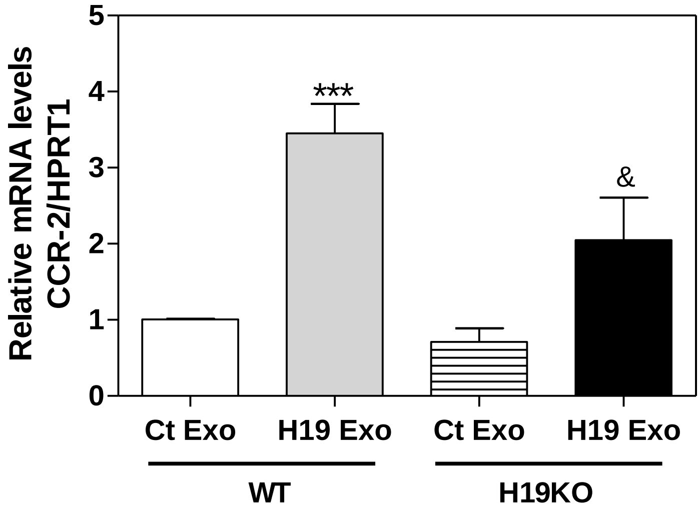

Supplementary figure 2

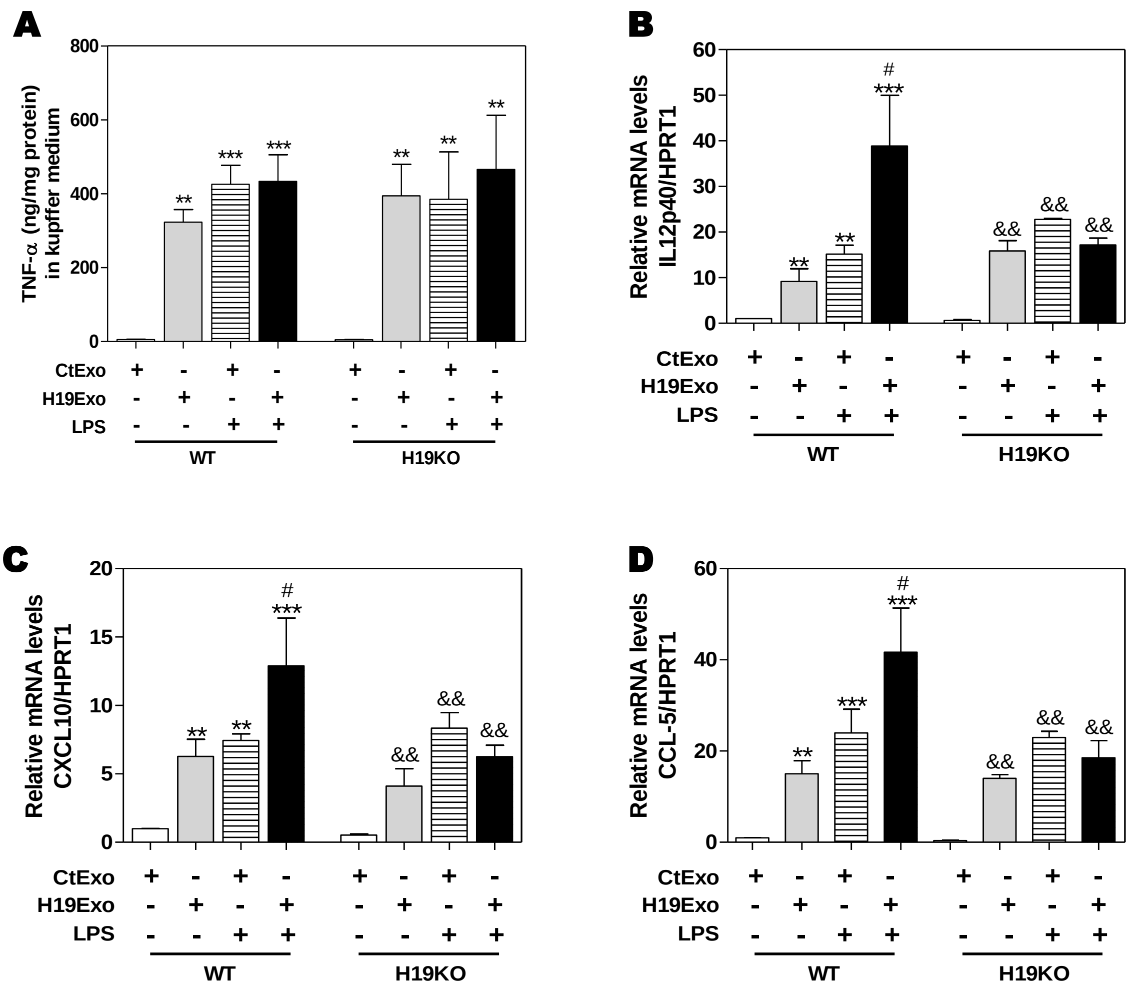

Supplementary figure 3

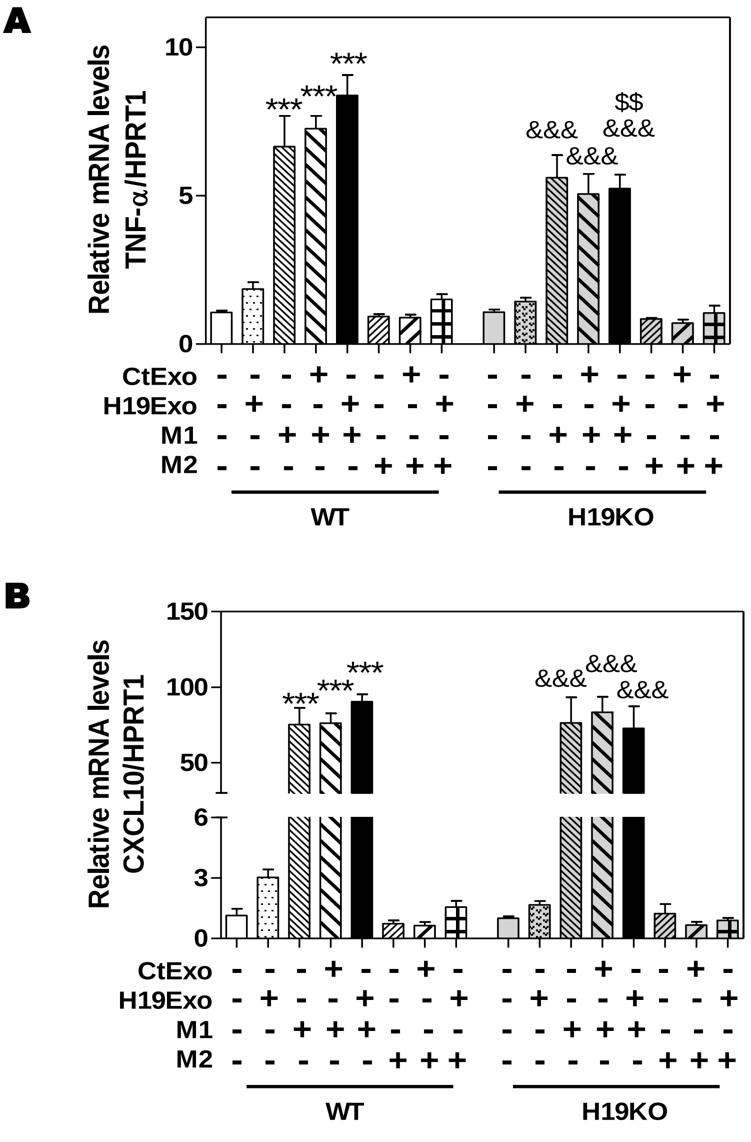

Supplementary figure 4

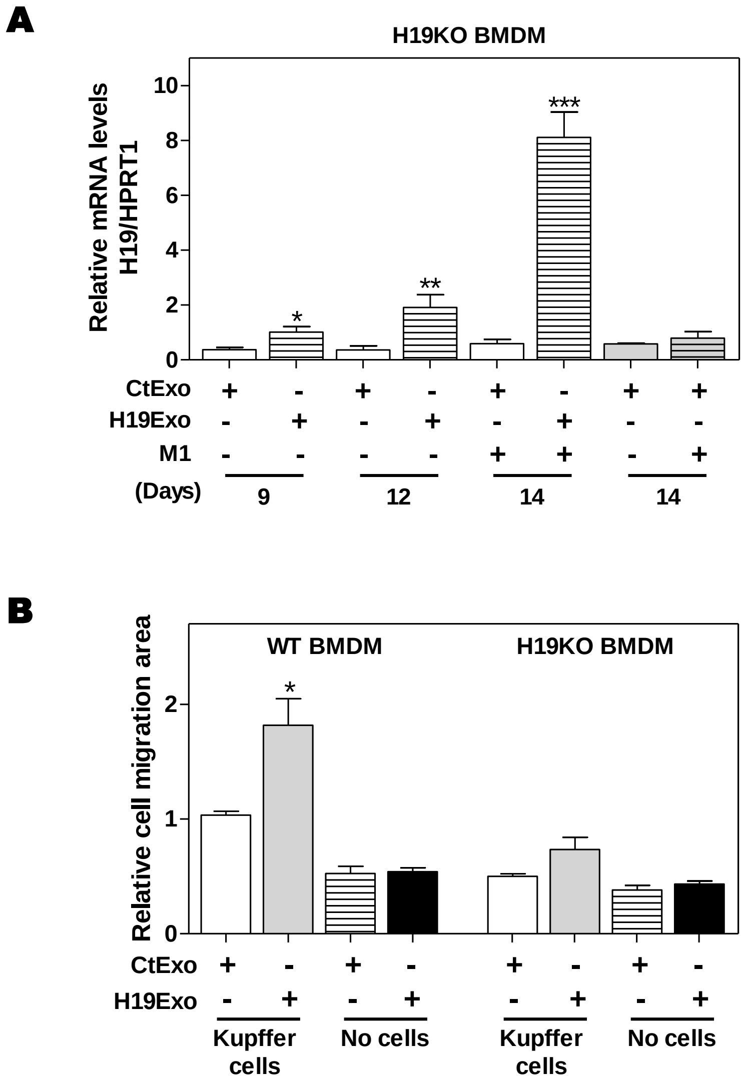

Supplementary figure 5

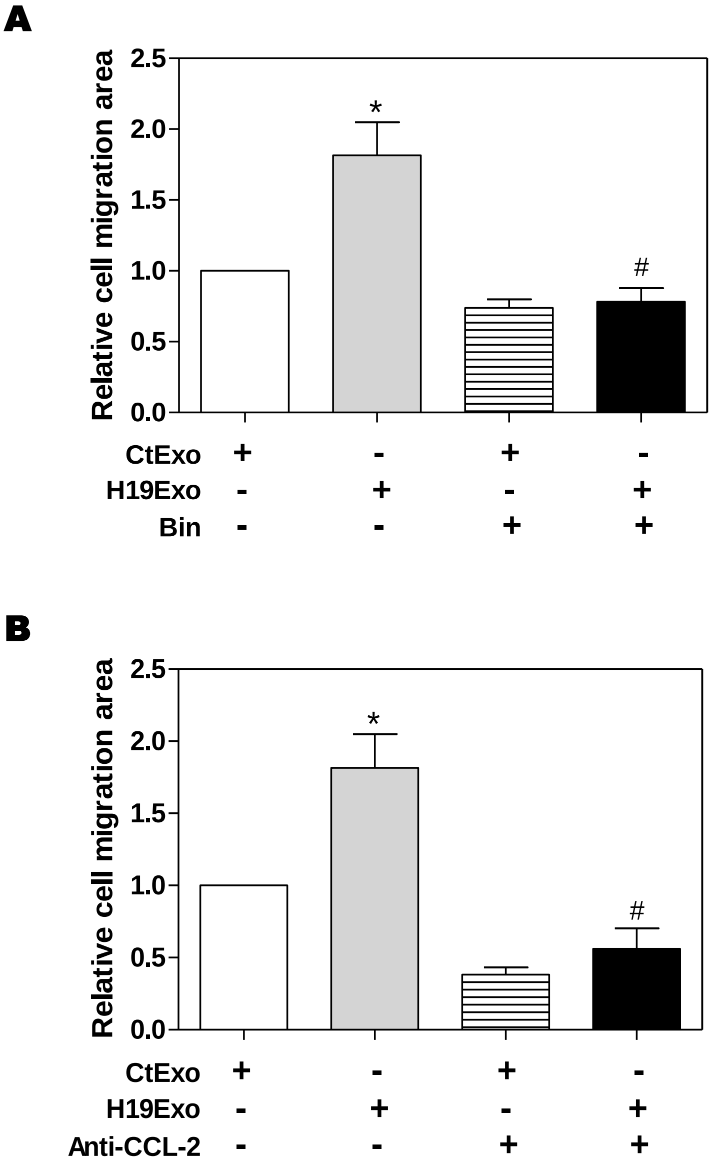

Supplementary figure 6

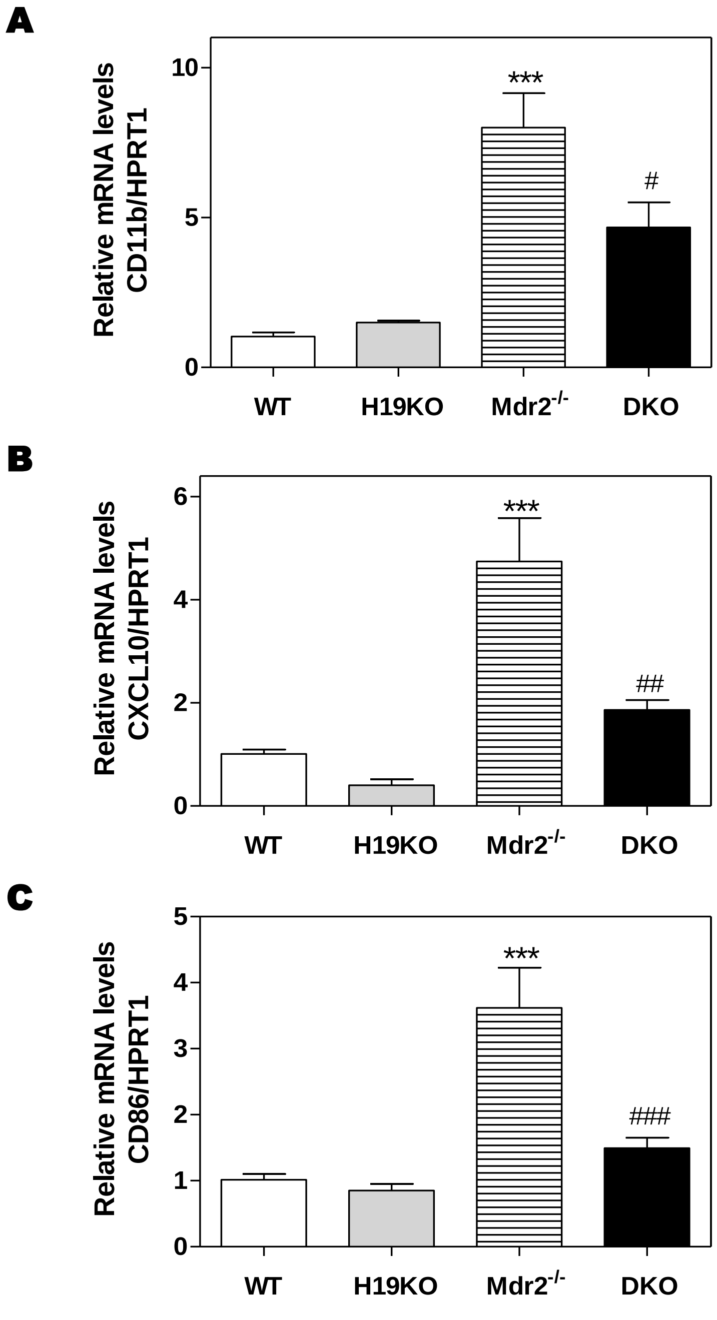

Supplementary figure 7

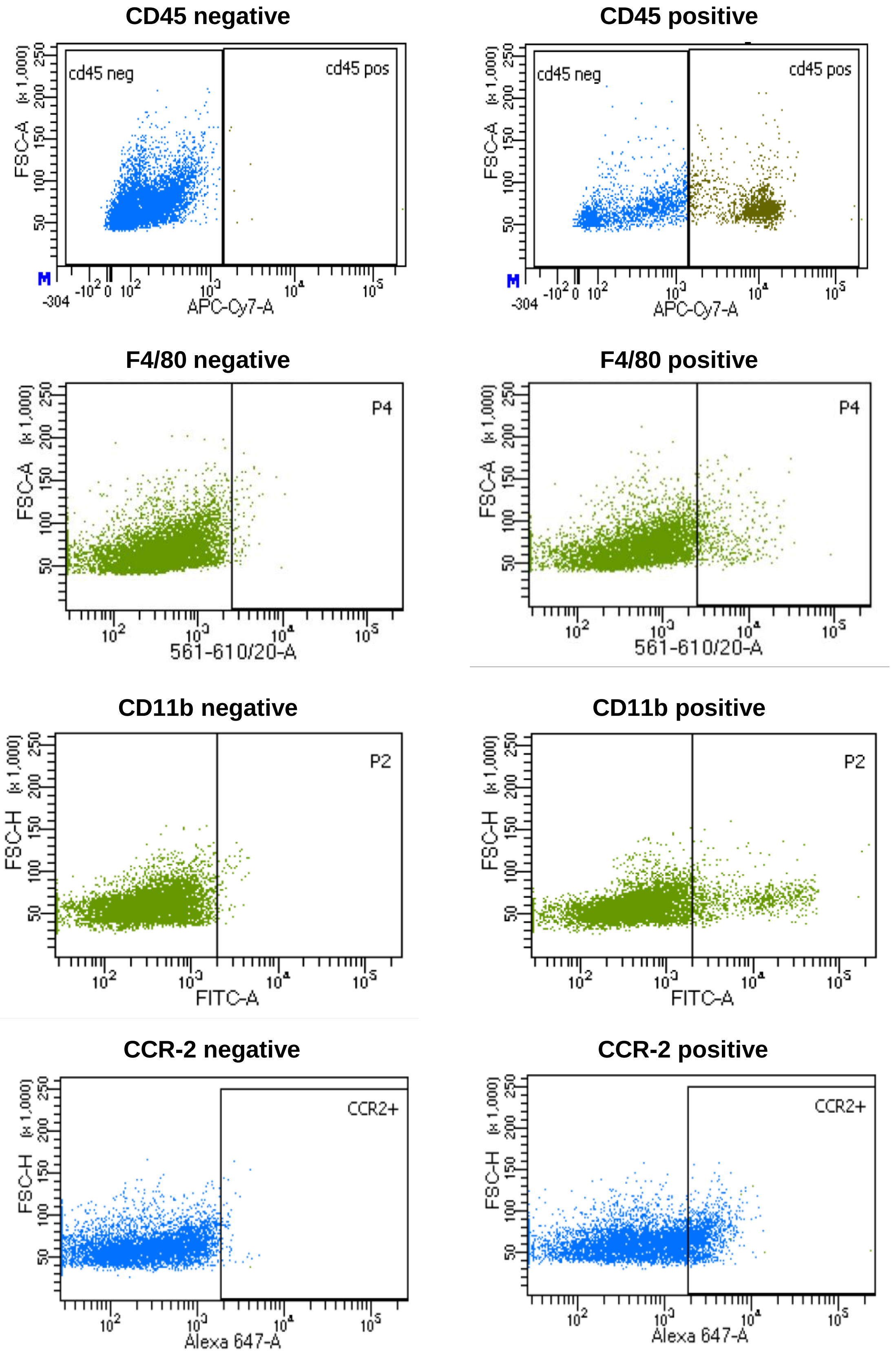

Supplementary figure 8

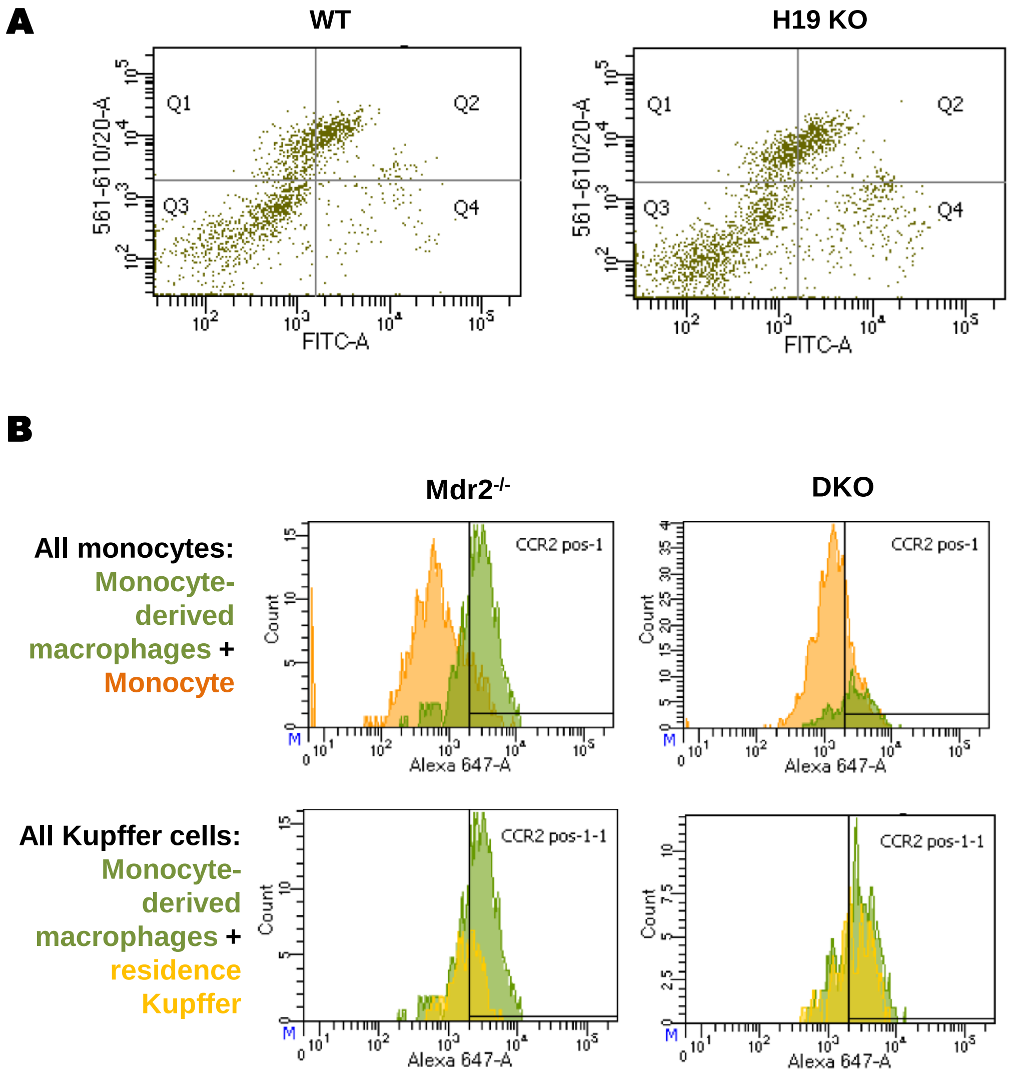

Supplementary figure 9

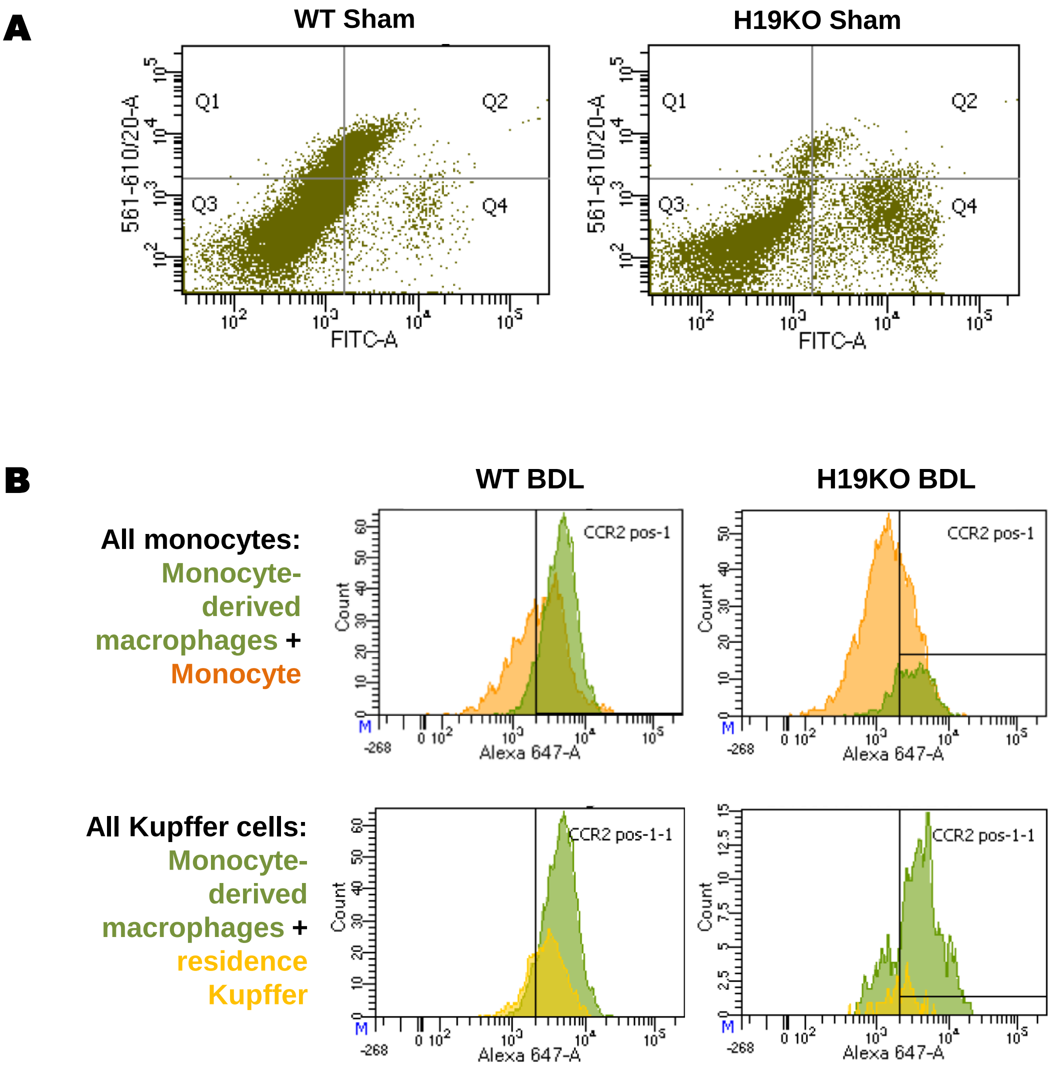

Supplement: Supplementary file 1 [file cells-09-00190-s001.pdf]
